# Dysregulated G2 phase checkpoint recovery pathway reduces DNA repair efficiency and increases chromosomal instability in a wide range of tumours

**DOI:** 10.1038/s41389-021-00329-8

**Published:** 2021-05-15

**Authors:** Madushan Fernando, Pascal H. G. Duijf, Martina Proctor, Alexander J. Stevenson, Anna Ehmann, Shivam Vora, Dubravka Skalamera, Mark Adams, Brian Gabrielli

**Affiliations:** 1 Mater Research Institute-The University of Queensland, Brisbane, QLD Australia; 2 Institute of Health and Biomedical Innovation, Queensland University of Technology (QUT), Faculty of Health, School of Biomedical Sciences, Brisbane, QLD Australia; 3 Centre for Data Science, Queensland University of Technology (QUT), Brisbane, QLD Australia; 4 University of Queensland Diamantina Institute, The University of Queensland, Translational Research Institute, Brisbane, QLD Australia

**Keywords:** DNA replication, Oncogenes

## Abstract

Defective DNA repair is being demonstrated to be a useful target in cancer treatment. Currently, defective repair is identified by specific gene mutations, however defective repair is a common feature of cancers without these mutations. DNA damage triggers cell cycle checkpoints that are responsible for co-ordinating cell cycle arrest and DNA repair. Defects in checkpoint signalling components such as ataxia telangiectasia mutated (ATM) occur in a low proportion of cancers and are responsible for reduced DNA repair and increased genomic instability. Here we have investigated the AURKA-PLK1 cell cycle checkpoint recovery pathway that is responsible for exit from the G2 phase cell cycle checkpoint arrest. We demonstrate that dysregulation of PP6 and AURKA maintained elevated PLK1 activation to promote premature exit from only ATM, and not ATR-dependent checkpoint arrest. Surprisingly, depletion of the B55α subunit of PP2A that negatively regulates PLK1 was capable of overcoming ATM and ATR checkpoint arrests. Dysregulation of the checkpoint recovery pathway reduced S/G2 phase DNA repair efficiency and increased genomic instability. We found a strong correlation between dysregulation of the PP6-AURKA-PLK1-B55α checkpoint recovery pathway with signatures of defective homologous recombination and increased chromosomal instability in several cancer types. This work has identified an unrealised source of G2 phase DNA repair defects and chromosomal instability that are likely to be sensitive to treatments targeting defective repair.

## Introduction

Cells are constantly exposed to stresses that produced DNA damage such as single or double strand breaks. To ensure the fidelity and integrity of the genome, mechanisms to detect and repair DNA damage are co-ordinated by cell cycle checkpoints. These delay cell cycle progression to allow time for repair to occur before allowing cell division and regulate DNA repair processes. The checkpoint mechanism activated is dependent on the type of DNA damage incurred^1^. Ataxia-telangiectasia-mutated (ATM) is activated in responses to double strand breaks (DSBs) whereas ataxia telangiectasia and Rad3-related (ATR) is activated by the presence of single stranded DNA (ssDNA). These related proteins play a central role in cell cycle checkpoints in eukaryotic cells by regulating proteins that signal checkpoint arrest, DNA repair, transcription and apoptosis^1^.

The G2 phase checkpoint is particularly important as the presence of DNA damage in mitosis may lead to aneuploidy and propagation of mutations to progeny^2^. When DNA damage is detected during G2 phase ATM/ATR kinases activate the checkpoint kinases CHK2 and CHK1, respectively to block the cell cycle entry into mitosis^3–5^. Once damage is repaired checkpoint signals are switched off to resume cell cycle; a process termed as checkpoint recovery. PLK1 is an essential mitotic kinase that regulates mitotic entry and progression^6^, and plays a pivotal role in G2 phase checkpoint recovery^7^. During G2 phase arrest, PLK1 is inactivated by dephosphorylation of Thr210 by the phosphatase PP2A/B55α^8^. Moreover, BORA, which is an AURKA cofactor required to promote PLK1 activation, and is targeted for degradation by SCF-βTrCP1 following phosphorylation by ATR^9^. Thus, inactivation of the AURKA-PLK1 axis is critically important to enforce G2 checkpoint arrest^10^.

ATM also regulates DSB repair through non-homologous end joining in G1 phase and homologous recombination repair (HRR) in S and G2 phase^11–13^. Therefore, bypassing the ATM-dependent checkpoint arrests by over-activation of PLK1 may impair these DNA damage repair mechanisms. Over-expression and activation of AURKA and inactivating mutations of *PPP6C*, the catalytic subunit of PP6, a negative regulator of AURKA activity, have been reported to disrupt HRR^14–16^. Downregulation of *PPP2R2A*/B55α regulatory subunit of PP2A that can dephosphorylate and downregulate PLK1 activity^8^ has also been reported to disrupt HRR^17^. PLK1 is reported to phosphorylate and inhibit the activity of MRE11 and 53BP1^14,18^. However, it remains unclear whether these roles for AURKA or PLK1 to downregulate HRR occur during G2 phase or mitosis. Moreover, how disruption of HRR is connected to bypass of the G2 phase checkpoint is also unknown.

Defective HRR is being exploited as an anti-cancer target using drugs such as the poly-ADP-ribose polymerase inhibitors (PARPi), although these are currently being used primarily in breast and ovarian cancers with mutations or loss of expression of *BRCA1/2* and a small set of HRR genes^19^. It is increasingly clear that PARPi sensitivity extends beyond defects in this narrow set of genes even in these two cancers that have relatively high frequency of HRR gene mutations^20,21^. Thus, new pathways that influence HRR may act as markers of sensitivity to drugs targeting defective HRR. Although individual components of the G2 phase checkpoint recovery pathway have been shown to have effects on DNA repair in model systems^14–16,18^, the mechanism by which this dysregulation effects the checkpoint, which checkpoint response is affected, and the outcome and occurrence of pathway dysregulation in cancers has not been established. Here we have investigated the effect of dysregulation of individual components of the AURKA-PLK1 checkpoint recovery pathway on PLK1 activity and their ability to bypass the G2 phase cell cycle checkpoint arrest, effects on DNA repair efficiency and genomic stability.

## Results

### Over-expression of AURKA overcomes the ATM-dependent G2 phase checkpoint arrest

To investigate the defective ATM checkpoint function produced by dysregulated AURKA- PLK1 pathway, we stably over-expressed wild type AURKA in two ATM checkpoint functional melanoma cells lines, A2058 and A375. AURKA was strongly over-expressed in both A2058 and A375 cells in all cell cycle phases compared to empty vector (EV) control cells (Supplementary Fig. S1A). Phosphorylation of AURKA Thr288 (pAURKA), an autophosphorylation site, was observed in AURKA over-expressing cells in all cell cycle phase, although this was reduced in the thymidine treated S phase arrest cells and was increased to a similar level in EV in mitotically arrested cells. AURKA over-expression had no effect on normal cell cycle progression of thymidine synchronised cell population, although it slowed transit through mitosis (Supplementary Fig. S1B–D).

**Fig. 1:**
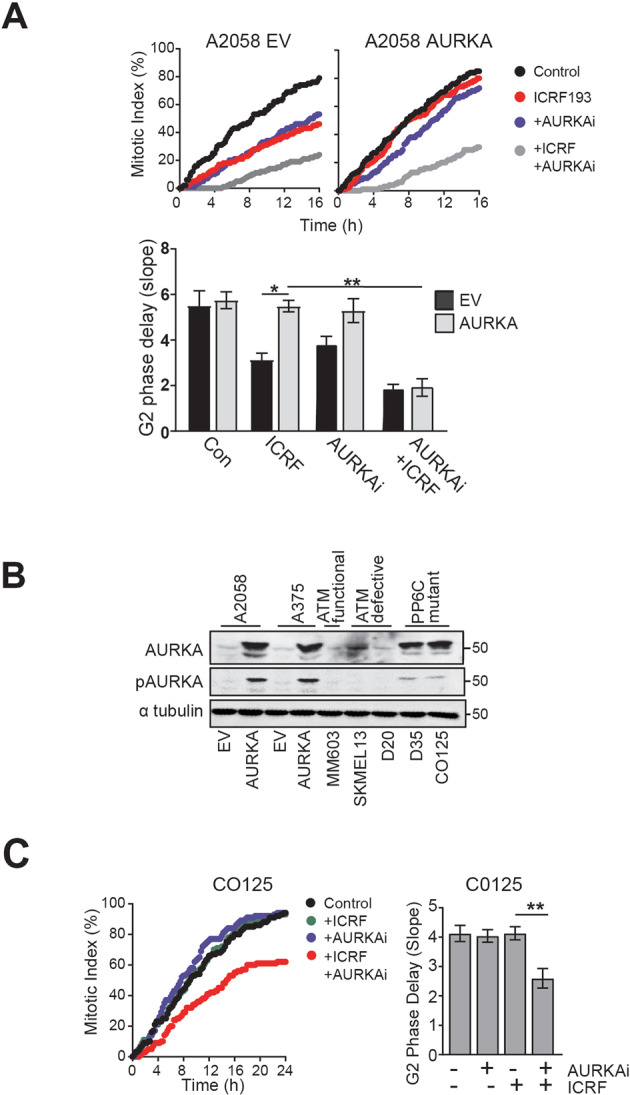
AURKA over-expression overcomes the ATM-dependent G2 phase checkpoint arrest. **A** A2058 EV and AURKA overexpressing cells were treated with either 5 μM ICRF193 or DMSO control. 200 cells were counted for each experimental group. Cumulative mitotic index was assessed as an indicator of G2 phase delay. The bar graph shows G2 phase delay quantified as the slope of the cumulative mitotic index curve using ICRF193 and 1 μM AURKA inhibitor (AURKAi) MK5801, as single and combination treatments, or DMSO. **B** Immunoblot of the level of AURKA and pAURKA in a panel of melanoma cell line (MM603 is ATM checkpoint functional, SKMEL13, D20 are ATM checkpoint defective, C0125 and D35 are PP6C mutants). α-tubulin was used as a loading control. **C** Bar graphs of the G2 phase delay of C0125 cells treated with ICRF193, AURKAi alone, or in combination. Bars represent means of three independent experiments (±SD); **p < 0.01; *p < 0.05.

AURKA over-expression overcame the ATM-dependent G2 phase checkpoint arrest triggered by the topoisomerase II catalytic inhibitor ICRF-193^22^. Time lapse imaging was used to assess the delay in mitotic entry (Fig. 1A). The slope of the cumulative mitotic index curve quantified the G2 phase delay (Supplementary Fig. S2A). ICRF193 transiently delayed mitotic progression in both A2058 and A375 EV lines but not AURKA over-expressing lines. The G2 phase delay was restored in AURKA over-expressing cells with the AURKA specific inhibitor MK5108 (Fig. 1A). AURKA over-expression modestly reduced the level of phospho-CHK2 Thr68 (pCHK2), a direct substrate of ATM, following treatment with ICRF193 or etoposide (Supplementary Fig. S2B).

The level of AURKA and pAURKA in the over-expressing cells was significantly higher than in other melanoma cell lines previously characterised as having an ATM checkpoint defect, although it was comparable to that in two melanoma cell lines with inactivating mutations in PP6C, a negative regulator of AURKA^16,23^ (Fig. 1B). The increased AURKA activity in the PP6C mutant cell lines drove bypass of the ATM-dependent checkpoint arrest triggered by ICRF193 in both mutant cell lines, and the delay was reinstated using selective inhibitors of AURKA (Fig. 1C; Supplementary Fig. S3). Taken together, these data demonstrate that activation of AURKA is sufficient to abrogate ATM-dependent G2 phase checkpoint.

### AURKA-dependent PLK1 activation is responsible for G2 phase checkpoint bypass

**Fig. 2:**
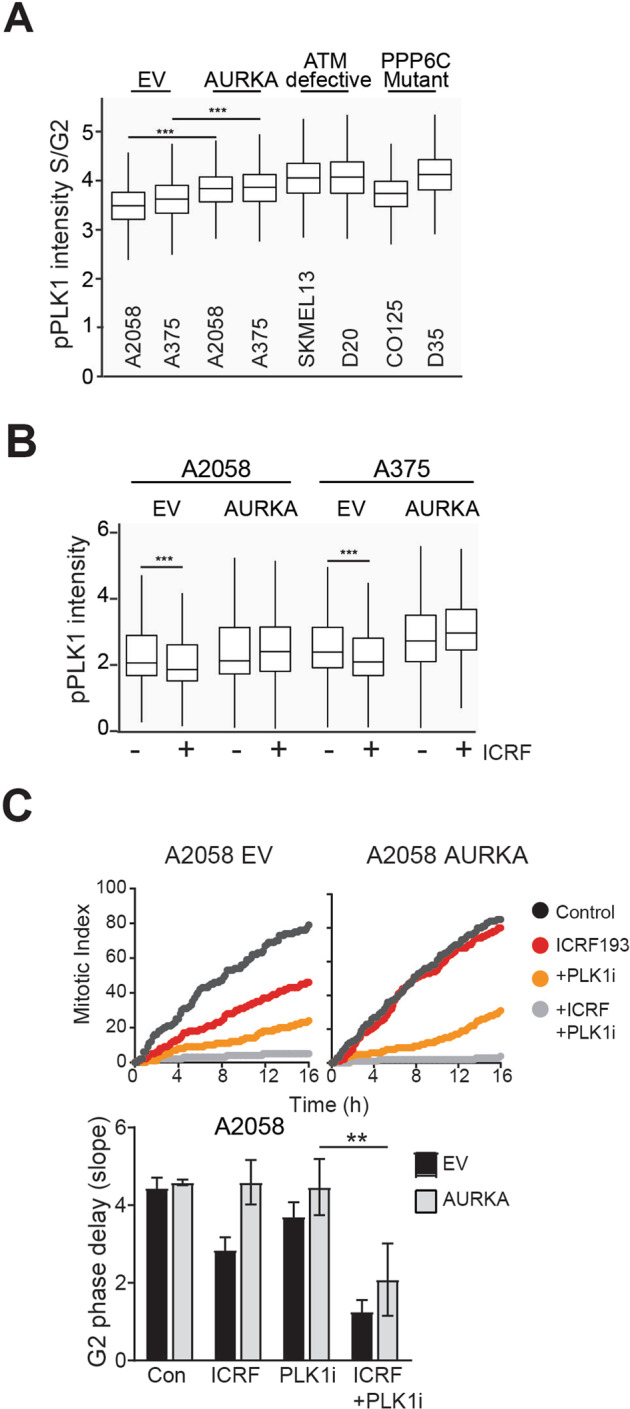
AURKA dependent PLK1 activation is required to overcome ATM dependent ATM checkpoint arrest. **A** Levels of pPLK T210 in S/G2 phase cells in a panel of asynchronously growing melanoma cell line (A2058 and A375 are ATM functional, SKMEL13 and D20 are ATM checkpoint defective, CO25 and D35 are PP6C mutants) were quantified on the basis of DNA content (DAPI) using high content imaging. **B** Box and whisker plot shows the level of pPLK1 in A2058 and A375 (EV and AURKA over-expressing) cell lines by high-content imaging. Cells were treated with or without ICRF193 for 6 h before fixing. Experiment was performed in triplicate. **C** G2 phase delay assessed by time lase imaging of A2058 EV, AURKA-overexpressing cells treated with ICRF193 or 100 nM PLK1 inhibitor (PLK1i) BI2536 alone, or in combination. Bars represent means (±SD) of three independent experiments. **p < 0.01; ***p < 0.001; ****p < 0.0001.

AURKA phosphorylates and activates PLK1 at Thr210 (pPLK1)^10,24^. Increased pPLK1 Thr210 was readily observed by immunofluorescence of AURKA over-expressing cells (Supplementary Fig. S4A). The specificity of the immunofluorescence signal was confirmed using two AURKA inhibitors which reduced pPLK1 signal to control levels (Supplementary Fig. S4B). Using high content imaging we could identify S/G2 phase cells on the basis of their DNA content. The level of pPLK1 was significantly higher in S/G2 phases in both AURKA over-expressing cell lines, and similarly elevated in ATM defective and *PPP6C* mutant melanoma cells (Fig. 2A).

**Fig. 3:**
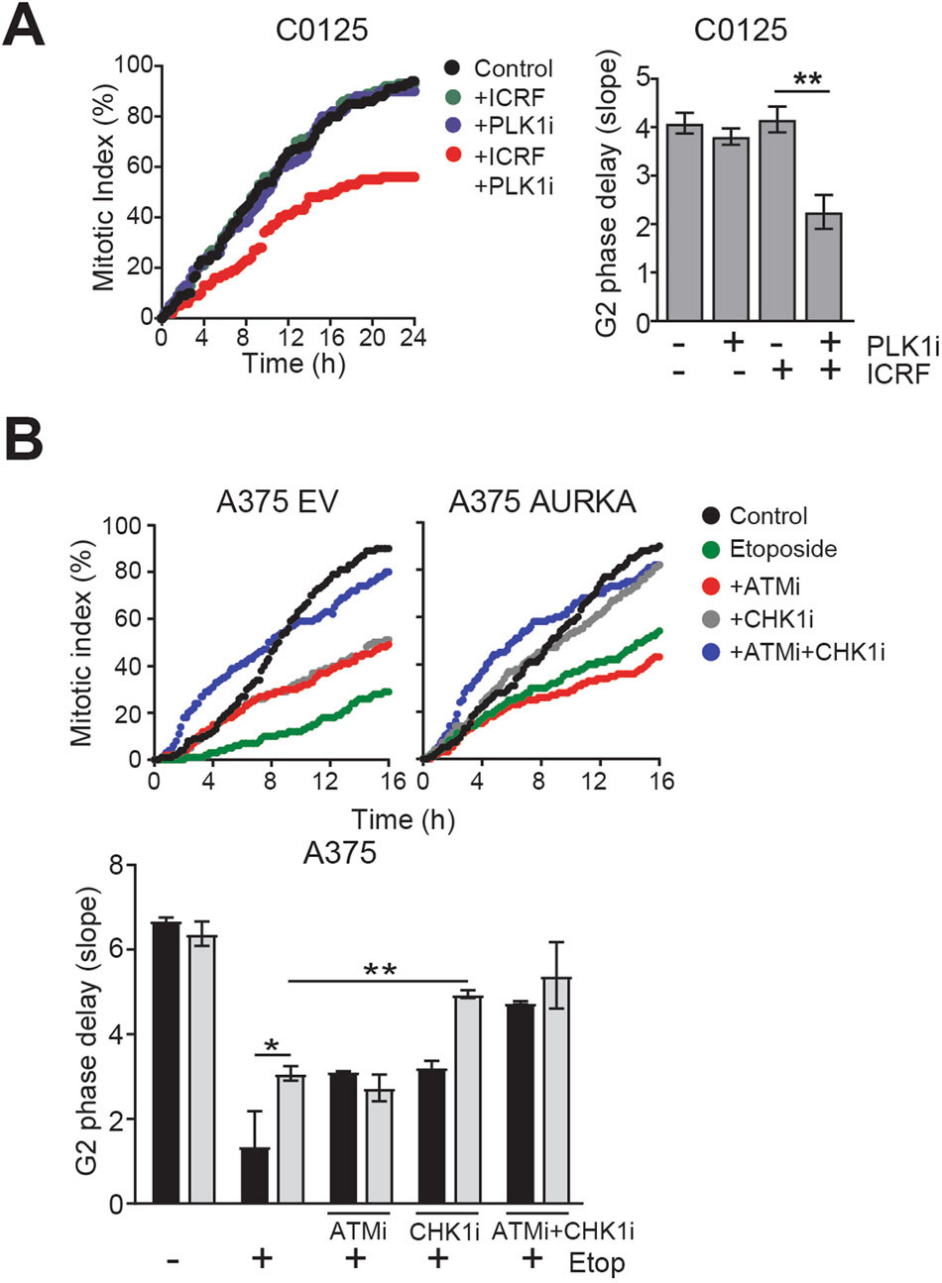
AURKA over-expression selectively overcomes ATM- but not ATR-dependent G2 phase checkpoint arrest. **A** G2 phase delay assessed by time lase imaging of PPP6C mutant C0125 cells treated with ICRF193 or 100 nM PLK1 inhibitor (PLK1i) BI2536 alone, or in combination. Bars represent means (±SD) of three independent experiments. **p < 0.01. **B** Representative line graphs demonstrate the rate of cells entering into the mitosis. A375 EV and AURKA overexpressing cells were treated with 4 μM of etoposide or 5 μM of ATM inhibitor (ATMi) KU55933 or 0.5 μM of CHK1 inhibitor (CHK1i) GNE323 alone, or in combination. Statistical analysis was performed using one-way ANOVA. Bars represent means (±SD); *p < 0.05.

ICRF193 treatment reduced the level of pPLK in EV but not AURKA over-expressing cells (Fig. 2B). This was confirmed by immunoprecipitation of PLK1. pPLK1 was detected following from immunoprecipitation of total PLK1 from mitotic cell lysates, and ICRF193 reduced the level of pPLK1 from EV cells but not AURKA over-expressing cells (Supplementary Fig. S4C, D). The contribution of PLK1 activity to ICRF193-induced G2 delay was demonstrated using the PLK1 inhibitor BI-2536 which alone induced a modest G2 phase delay in only the EV cells. When combined with ICRF193, it enhanced the G2 phase delay in EV cells and restored the delay in AURKA over-expressing cells (Fig. 2C). PLK1 inhibition also restored the ICRF193 G2 phase in the *PPP6C* mutant C0125 cells (Fig. 3A). This demonstrated that AURKA over-expression or PP6C mutation drives premature exit from the ATM checkpoint by maintaining PLK1 activation.

### AURKA over-expression selectively overcomes ATM but not ATR G2 phase checkpoint arrest

Etoposide activates ATM and ATR dependent checkpoint signalling and produced a strong G2 phase delay in both EV A2058 and A375 cell lines. AURKA over-expression only partially attenuated this delay (Fig. 3B, Supplementary Fig. S5). We reasoned that the strong G2 phase delay was due to simultaneous activation of ATM and ATR checkpoint signalling whereas ICRF193 only activates ATM checkpoint signalling. Inhibitors of ATM (KU55933) or CHK1 (GNE-323) partially attenuated the etoposide induced G2 phase delay in EV cells (Fig. 3B). Inhibition of ATM had no effect on CHK1 activation (Supplementary Fig. S6A), or on the G2 phase delay in AURKA over-expressing cells, whereas CHK1 inhibition effectively abrogated the delay in the AURKA over-expressing cells (Fig. 3B). Inhibition of both ATM and CHK1 pathways completely abrogated the etoposide induced arrest in EV cells.

UVR activates an ATR-CHK1-dependent G2 phase checkpoint arrest^25^. Irradiation of the isogenic cell lines with ultraviolet radiation (UVR; 150 Jm^2^) activated CHK1 (pCHK1 S317) in both EV and AURKA over-expressing cell lines to a similar level (Supplementary Fig. S6B). Thymidine arrest also activates an ATR-CHK1 dependent checkpoint arrest, but AURKA over-expression was ineffective in overcoming this checkpoint indicated by the similar degree of synchrony obtained in EV and AURKA over-expressing cells (Supplementary Fig. S1B, C). These data demonstrate that AURKA over-expression effectively overcomes the ATM but not ATR-dependent G2 phase checkpoint arrest.

### Depletion of PPP2R2A/B55α overcomes G2 phase Etoposide triggered checkpoint arrest

**Fig. 4:**
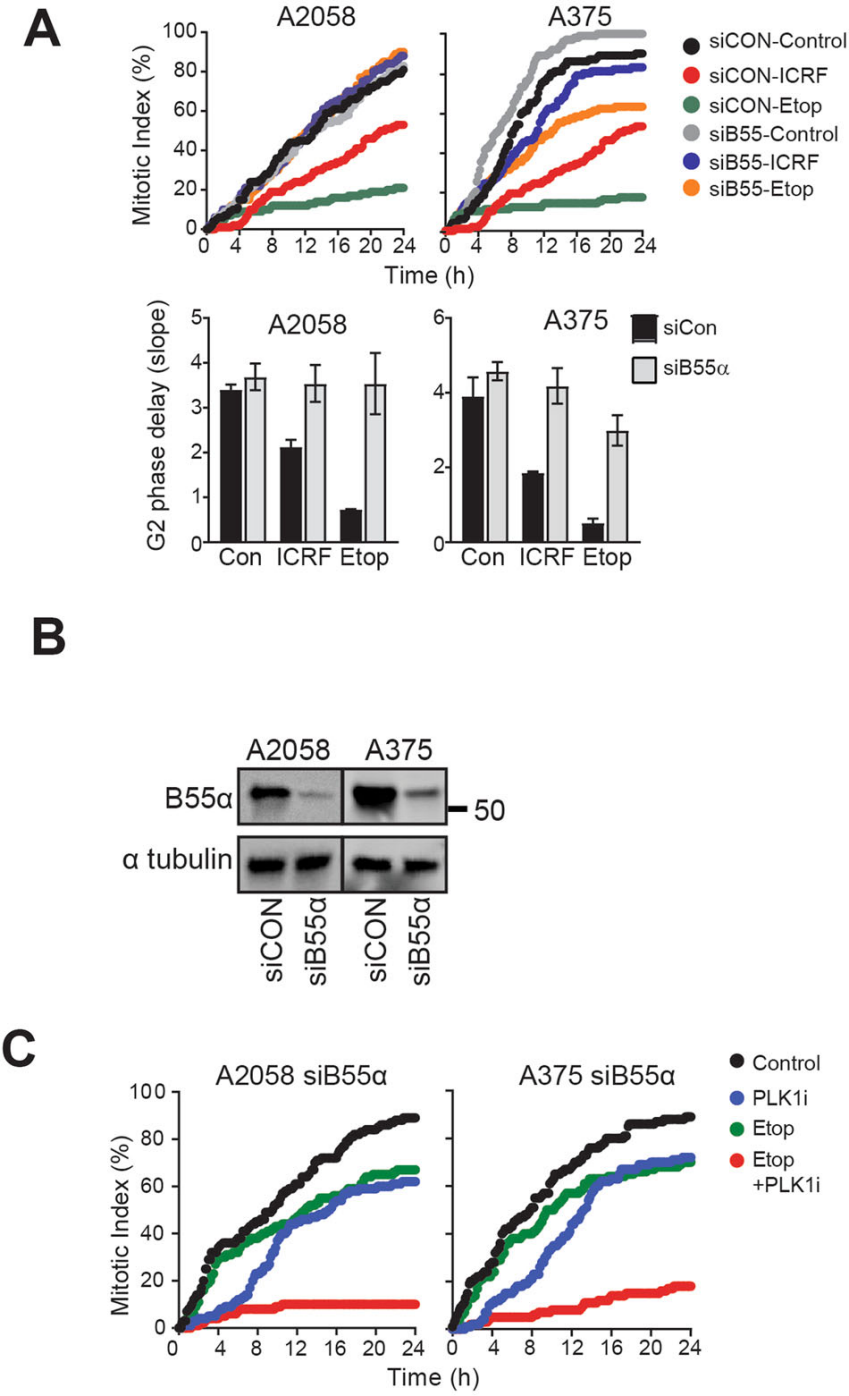
Loss of *PPP2R2A*/B55α overcomes Etoposide induced G2 checkpoint arrest. Asynchronously growing A2058 and A375 cells were transfected with either control or B55α siRNA. **A** Transfected cells were treated with 5 μM ICRF193 or 2 μM etoposide, and the G2 phase delay was quantified using time lapse imaging. The data are from three independent experiments. **B** Cell lysates were immunoblotted with B55α. α-Tubulin was used as a loading control. **C** siB55α transfected cells were treated with 2 μM etoposide, 500 nM BI2536 alone or in combination. G2 phase delay was quantified using time lapse imaging.

*PPP2R2A*/B55α is a targeting subunit of PP2A that has been shown to selectively promote entry into mitosis from the G2 phase checkpoint arrest^26,27^. When *PPP2R2A* was depleted using siRNA it was found to completely abrogate the ICRF193 and etoposide induced G2 in both cell lines (Fig. 4A, B). This effect was dependent on PLK1 as inhibition of PLK1 restored the strong G2 phase delay observed with etoposide treatment of the control siRNA treated cells (Fig. 4C; Supplementary Fig. S7). The data suggested that unlike AURKA over-expression or *PPP6C* mutation, regulation of PLK1 by PP2A can overcome the ATM-dependent checkpoint induced by ICRF193 and both ATM and ATR-dependent checkpoint arrest induced by etoposide.

### Dysregulation of PP6C-AURKA-PLK1 pathway reduces DNA repair efficiency

**Fig. 5:**
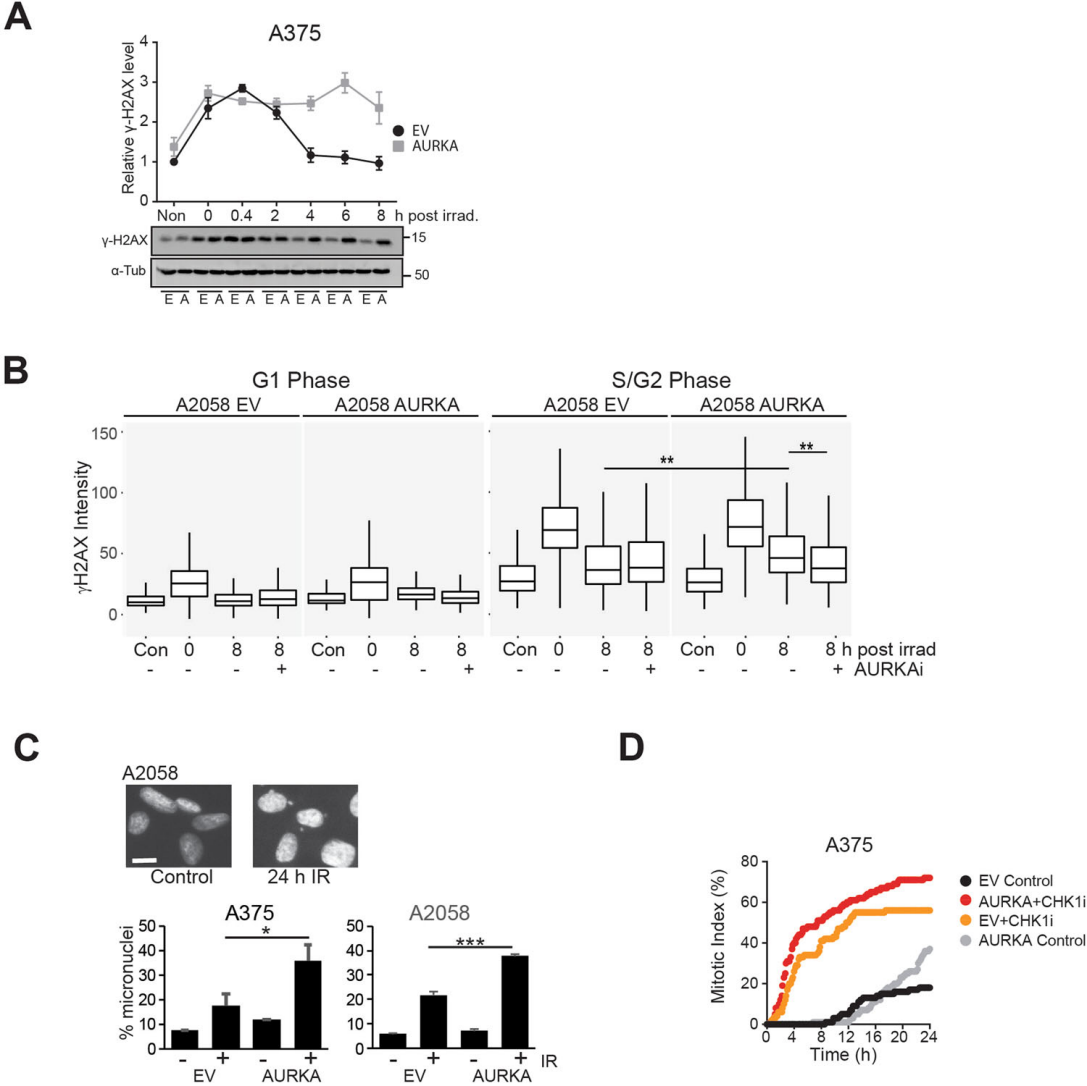
AURKA overexpression reduces HRR efficiency. **A** Time dependent immunoblot analysis for γH2AX levels and quantification. Asynchronous A2058 EV and AURKA overexpressing cells were exposed to γ radiation (10 Gy), harvested at indicated hours after exposure, and immunoblotted for γH2AX. α-Tubulin was used as a loading control. [B] DNA content (DAPI intensity) of cells determined by high content imaging. γ-H2AX intensities were plotted against DNA content. Using the DNA profile, G1 and S/G2 populations were identified. **B** Box and whisker plot shows γH2AX intensities in G1 and S/G2 cell cycle phases. A2058 EV and AURKA overexpressing cells were either fixed as unirradiated controls (Con) or irradiated with 6 Gy and fixed immediately after irradiation (0 h), or after 8 h either without or with AURKAi MK5108 (1 μM). DNA content (DAPI) and γ-H2AX intensities were quantified in G1 and S/G2 cell cycle phases using high content imaging. **C** Both EV and AURKA over-expressing lines were irradiated with 6 Gy radiation then cells were fixed 24 h later and stained for DNA. Inset shows the micronuclei formed. The percentage of cells with micronuclei was in triplicate samples for each condition and cell line. 300–1000 cells were counted for each replicate. **D** A375 EV and AURKA overexpressing cells were exposed to γ radiation (10 Gy), treated without or with CHK1i then followed by time lapse microscopy. The cumulative mitotic index for each culture was assessed.

To assess the effects of AURKA-PLK1 pathway dysregulation on DNA repair, cells were irradiated with 10 Gy gamma radiation and repair measured using γH2AX as a marker of DNA damage. γH2AX levels were maximal at 20 minutes after irradiation in both cell lines confirming that ATM dependent H2AX S139 phosphorylation is unaffected. Whereas levels declined in the empty vector cells, γH2AX remained elevated in the AURKA over-expressing cells (Fig. 5A). ATM regulates both G1 phase non-homologous end joining and S/G2 phase HRR. The cell cycle phase of cells was determined by DNA content and the level damage assessed by γH2AX staining intensity using high content imaging (Fig. 5B; Supplementary Fig. S8A). G1 phase damage was efficiently repaired in EV and AURKA over-expressing cells, whereas S/G2 phase damage was more efficiently repaired in EV controls (Fig. 5B, Supplementary Fig. S8B). Inhibition of AURKA significantly reduced the level of DNA damage in AURKA over-expressing but not in EV control cells (Fig. 5B, Supplementary Fig. S8B).

To demonstrate that γH2AX levels reflected the level of DNA damage, a micronucleus assay was performed. The micronuclei are fragments of chromosomes produced by unrepaired double strand breaks that fail to congress at metaphase, forming micronuclei in the subsequent interphase^28^. The proportion of cells with micronuclei increased 24 h after irradiation when cells had undergone only one mitosis, the level increased significantly in the AURKA over-expressing compared to the EV cells (Fig. 5C). Together, these data demonstrate that AURKA over-expression was selectively effecting S/G2 phase repair suggesting this was due to reduced HRR efficiency.

Co-staining for the S/G2 phase marker Cyclin A revealed that both EV and AURKA over-expressing cells accumulated in S/G2 phase (Supplementary Fig. S8C). This was confirmed by time lapse imaging of irradiated cells and was due to CHK1 activation, as inhibition of CHK1 resulted in rapid entry into mitosis (Fig. 5D). This indicated that the reduced repair efficiency was not a consequence of a reduced G2 phase arrest in the AURKA over-expressing cells, but was likely to be related to inappropriate AURKA-PLK1 activity.

These findings were validated in ATM checkpoint defective^29^ and *PPP6C* mutant cell lines. DNA damage was more efficiently repaired when AURKA or PLK1 was inhibited (Supplementary Fig. S9A, B). The radiation induced G2 phase delay was also observed in these cell lines indicated by the accumulation of Cyclin A staining cells (Supplementary Fig. S9C).

ATM is an integral regulator of DNA repair proteins such as BRCA1, MRN complex and 53BP1 by promoting their localisation to sites of DNA damage and activity^30^. Loss of ATM activity blocks the formation of foci for DNA repair proteins^31^ such as 53BP1 and RAD51 nucleofilaments^32^. AURKA over-expression did not affect irradiation induced RAD51 foci formation (Supplementary Fig. 10A, B), or localisation of RAD51 to the chromatin following DNA damage (Supplementary Fig. S10C). ATM-dependent 53BP1 foci were also found to be unaffected by AURKA over-expression (Supplementary Fig. S10D, E). By contrast, inhibition of ATM reduced γH2AX and RAD51 foci formation in both EV and AURKA over-expressing cell lines (Supplementary Fig. S10F), demonstrating that AURKA over-expression did not inhibit normal ATM-dependent responses to DNA damage such as RAD51 and 53BP1 focus formation.

### Dysregulation of checkpoint recovery pathway increases genomic instability across a range of cancers

**Fig. 6:**
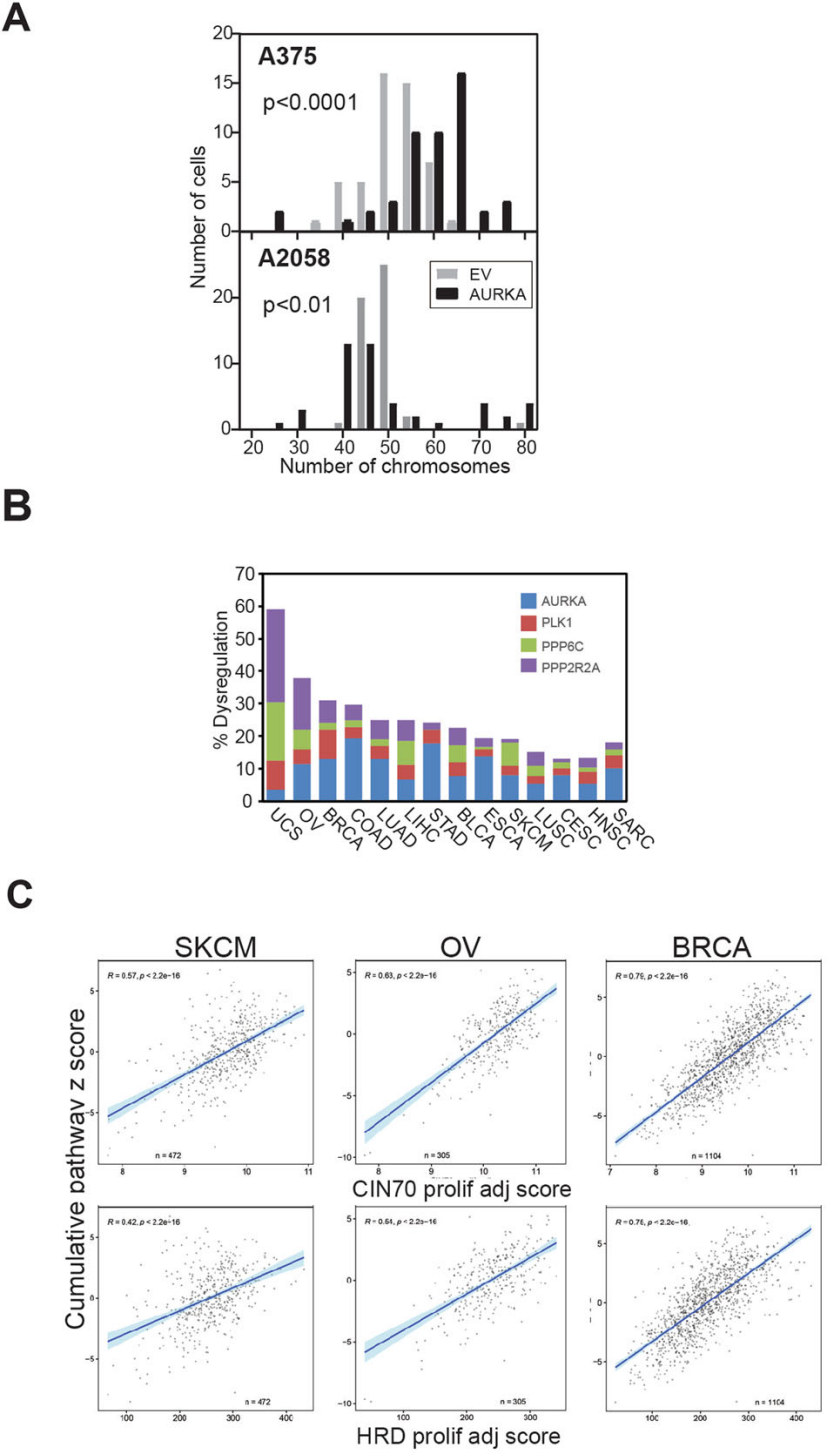
PP6C-AURKA-PLK1 dysregulation promotes chromosomal instability. **A** Histograms of the numbers of chromosomes in the isogenic A375 and A2058 pairs, counted in 50 mitotic chromosome spreads for each line. **B** The bars represent the cumulative dysregulation of *PPP6C*, *AURKA*, *PLK1* and *PPP2R2A*/B55α as a percentage of total cases in TCGA pan cancer data set. BLCA, bladder cancer; BRCA, breast cancer; LIHC, liver hepatocellular carcinoma; LUAD, lung adenocarcinoma; CESC, cervical squamous cell carcinoma; COAD, colorectal adenocarcinoma; ESCA, oesophageal adenocarcinoma; HNSC, head and neck SCC; Liver, Liver hepatocellular carcinoma; LUSC, lung SCC; OV, ovarian cancer; STAD, stomach adenocarcinoma; SKCM, melanoma; UCS, Uterine carcinoma; SARC, sarcomas. **C** Correlation of the cumulative pathway score for *PPP6C*-*AURKA*-*PLK1*-*PPP2R2A* and CIN70 and HRD proliferation adjusted (prolif adj) scores for the indicated tumour types from TCGA.

The decatenation checkpoint is uniquely ATM dependent^33^, and checkpoint defect increases genomic instability due to incomplete decatenation of replicated chromosomes prior to cell cycle progression^29^. Chromosome numbers from mitotic spreads of EV and AURKA overexpressing cells that had been passaged >8 times revealed that AURKA over-expression resulted in a significantly broader distribution of chromosome numbers than their isogenic EV lines (Fig. 6A; *p* > 0.01 Kolmogorov-Smirnov test).

PP6-AURKA-PLK1-B55α pathway dysregulation was assessed using the Cancer Genome Atlas (TCGA) dataset. Gene amplification and upregulation of *AURKA* and *PLK1* mRNA, truncating mutations in *PPP6C*, deep deletion and low mRNA levels of *PPP6C* and *PPP2R2A*/B55α were scored as indicators of pathway dysregulation. The appropriate dysregulated of these four genes (down-regulation of the phosphatases and up-regulation of the kinases) were found in 20% of melanomas, 40% of ovarian cancers, and up to 60% of uterine cancers (Fig. 6B). We further analysed the expression of these four genes by using summed z-scores of these four genes as a continuous score. This revealed a very strong correlation with established measures for chromosomal instability (CIN70 score^34^) and homologous recombination defects (HRD score^35^) across all investigated cancer types (Supplementary Fig. S11). A subset of the CIN70 and HRD signature include cell cycle genes, and it is well-established that cell cycle genes are often upregulated in tumours as a consequence of an increased proliferative index^36,37^. Therefore, we removed all cell cycle genes from the CIN70 and HRD signature scores to determine the proliferation adjusted “CIN70_prolif_adj” and “HRD_prolif_adj” signatures. Our cumulative pathway score remained strongly correlate the modified CIN70 and HRD scores (Fig. 6C), indicating that the strong correlations with chromosome instability and HR defects are not purely a result of associations with cell proliferation or overlapping gene sets, as none of our pathway genes are components of either score. Additional analysis of 250 random, non-overlapping four-gene sets using the same approach showed that the PP6C-AURKA-PLK1-B55α score correlation is significantly stronger and more significant than expected by chance, demonstrating the robustness of the correlations that we observed (Supplementary Fig. S12). These findings are strong evidence that dysregulation of the PP6C-AURKA-PLK1 checkpoint recovery pathway is a common feature of many cancers and strongly associated with defective G2 phase DNA repair and increased chromosome instability.

## Discussion

Here we showed that dysregulation of the PP6-AURKA-PLK1-B55α G2 phase checkpoint recovery pathway effectively bypasses the ATM-dependent G2 phase checkpoint arrest, reduced G2 phase DNA repair efficiency and increased chromosomal instability. The effect of AURKA over-expression was restricted to G2 phase ATM-dependent checkpoint activated cells, with few obvious effects observed in normal cell cycle progression into mitosis.

It was reported that AURKA over-expression did not activate PLK1 sufficiently to overcome a G2 phase checkpoint arrest except when expressed as a chimera with its co-factor BORA in U2OS cells^38^. AURKA over-expression was sufficient to block the G2 phase checkpoint inactivation of PLK1 in the two models used here, and co-expression of BORA had no effect on the ATR-dependent checkpoint (unpublished observations). The finding that depletion of the B55α subunit of PP2A can overcome both ATM and ATR-dependent G2 phase checkpoints may indicate that negative regulation of PLK1 by PP2A plays a larger role in controlling PLK1 activity in response ATR activation. We have previously found that depletion of B55α effectively overcomes the ATR-dependent G2 phase checkpoint arrest in response to ultraviolet radiation^26^.

AURKA-PLK1 dysregulation had no effect on either G1 phase repair of DSBs or S phase replication stress induced ATR-dependent checkpoint activation, but it reduced G2 phase DNA repair. Previous studies have reported that dysregulation of AURKA-PLK1 disrupted S/G2 phase HRR, indicated by reduced RAD51 foci after damage^15,16^. However, we failed to observe any defect in the ATM-dependent RAD51 or 53BP1 focus formation, further evidence that ATM activation was normal in response to damage. Further, the reduced repair was not a consequence of failure to cell cycle arrest as AURKA dysregulation only overcame the ATM-dependent checkpoint, the ATR-CHK1-dependent G2 phase checkpoint remained intact. PLK1 directly phosphorylates and inactivates MRE11, a component of the MRN complex thereby reducing repair^14^, and it is this direct inhibition of the repair machinery by PLK1 that is likely to be responsible the reduced repair observed.

Dysregulated AURKA-PLK1 increased chromosomal instability, a consequence of defective DNA repair and abrogation of the decatenation checkpoint. The accumulation of chromosomal number changes in both models suggests that the loss of the decatenation checkpoint is likely to be a significant contributor to the instability observed. Bypass of the checkpoint arrest would result in loss of large fragments or entire chromosomes^29^. Because ATM activation occurs normally in AURKA-PLK1 recovery pathway defective cells, pro-survival signals from ATM activated in response to DNA damage or catentated chromosomes such as NF-kB activation^39^ would be maintained, aiding the survival of daughter cells. This would provide an advantage compared with complete loss of function mutation of ATM and may explain the higher prevalence of this pathway dysregulation compared to ATM loss of function mutation.

We have found dysregulation of the PP6-AURKA-PLK1-B55α checkpoint recovery pathway in a wide range of tumours and correlated strongly with both the CIN70 and HRD scores in patient tumours. The defective G2 phase DNA repair suggests that tumours with this pathway defect might be more sensitive to PARP inhibitors, and this has been shown by targeted dysregulation of this pathway^14,15,17^. BRCA1 is involved in TOPOIIα-mediated decatenation^40^ and the chromosome copy number changes found in *Brca1* mutant tumours is reminiscent of AURKA over-expressing cells^41^. Thus, the defective checkpoint recovery pathway has many similarities to BRCA mutation providing further support for the use of PARP inhibitors to treat cancers with dysregulated PLK1 checkpoint recovery pathway.

This study has brought together disparate findings on the effects of AURKA, PPP6C, PLK1 and B55α-PP2A dysregulation and shown that they operate as a pathway controlling exit from the ATM-dependent G2 phase checkpoint arrest and regulate decatenation and G2 phase DNA repair. While these events are co-ordinated in normal G2 phase DNA damage response, dysregulation of any one of the components of this pathway will reduce the efficiency of G2 phase DNA repair and decatenation and increased chromosome instability. Dysregulation of this pathway is a common feature of a wide variety of cancers and represents an unrealised source of G2 phase DNA repair defect that may be targeted in manner similar to current approaches used to target BRCA mutant HRR defective tumours.

## Materials and methods

### Cell lines and culturing conditions

The human melanoma cell lines (A2058, A375, MM603, D20, SKMEL13, CO25 and D35) were cultured in in RPMI 1640 media (Invitrogen, Mulgrave, Vic., Australia) containing 10% Serum Supreme (Lonza BioWhittaker, Basel, Switzerland), 2.5 mM HEPES (Invitrogen), 1 mM sodium pyruvate (Gibco) and 2 mM L-glutamine (Gibco). All cell lines were confirmed by STR profiling and to be mycoplasma free. Cells were synchronised using a single thymidine block (2.5 mM thymidine for 24 h), plates were then washed three times with pre-warmed PBS then released into fresh media supplemented with 24 μM thymidine and deoxycytidine.

### AURKA over-expression in melanoma cell lines

The lentiviral expression clone AURKA-pLV411 was generated using the pLV411 lentiviral vector using Gateway LR recombinase (Invitrogen) as described previously^42^. After lentiviral transduction, A2058 and A375 cells sorted by FACS for their GFP signal.

### Time-lapse microscopy

Cells were seeded in multi well plates and time lapse imaging was performed using an Olympus IX81 Live imaging microscope as described previously^22^. For inhibitor studies, cells were irradiated, or drug treated immediately prior to starting imaging. Images were captured at 10 min intervals for 16 h to 24 h. Cell morphology was monitored to observe entry into mitosis. Time required to entry into mitosis was recorded. Minimum 200 cells from each treatment were monitored to record mitotic entry. The rate of cells entry into the mitosis was represented as cumulative mitotic index. Slope of the cumulative mitotic index curve was quantified as a measure of G2 phase delay, calculated as *Slope* = *(y−c)/x* were y is the Y axis coordinate, x is the X axis coordinate and C is the Y axis intercept. An example of the slope measurement is shown in Supplementary Fig. 2A.

### Immunoblotting

Cell pellets were lysed and prepared for immunoblotting as previously^29,43^. Membranes were probed with antibodies against phospho-AURKA T288 #3079, phospho-CHK2 T68 #2661, phospho-CHK1 Ser317 #2344, phospho-H2AX Ser139 #2577, B55α #2290, Histone 3 #9715 (Cell Signalling), AURKA (BD Bioscience #610938), PLK1 (Millipore) #05-844, Rad51 (14B4) # NB100-148 (Novus) and α-tubulin (Abcam). Proteins were visualized using chemiluminescence detection (Fusion SL Viber Lourmat imaging system). Protein levels were quantified using IMAGEJ software (http://rsbweb.nih.gov/ij/).

### Immunofluorescence

Cells grown on poly-L-lysine-coated glass coverslips were fixed with 4% formaldehyde (Sigma) and permeabilized with 0.1 % Triton X-100 in PBS. To visualize Rad51 foci, permeabilized cells were incubated with 1000 U/ml DNAse (Sigma) diluted in DNAse binding buffer (50 mM Tris/HCl, I0 mM MgCl2, 0.75% BSA, pH 7.5) for 30 min at 37 °C. Cells were incubated with 3% BSA to block. Cells were immunostained with antibodies against human pPLK1 #9062, phospho-H2AX (Cell Signalling) and 53BP1 #612522 (BD Biosciences), RAD51 (14B4) # NB100-148 (Novus), 4,6-diamidino-2-phenylindole (DAPI) DNA stain, and relevant secondary antibodies.

### High content imaging

Cells were seeded in black wall clear-bottom 96-well viewplates (Costar). Cells were fixed, permeabilised and blocked as for immunofluorescence and immunostained with appropriate primary and secondary antibodies. DNA was stained with DAPI. Images were acquired by InCell 6500HS analyser, using 20x objective. Images were processed using CellProfiler Version 2.2.0 (Carpenter Lab, Broad Institute of MIT and Harvard). A minimum of 2000 cells were acquired and analysed. The images and data were exported to R software (R studio 3.4.0) for statistical analysis.

### Chromosome spreads

Cells were treated with 0.5 μg/ml of nocodazole for 4 h. Mitotic cells were harvested by mechanical shake-off and suspended in a hypertonic buffer containing 75 mM KCl for 16 min. Cells were then fixed with methanol (3:1 solution of methanol: glacial acetic acid), washed several times in fixative then resuspended in a small volume of fixative. Cells were drawn into a plastic transfer pipet tip and released from 6 inches height above onto a pre-chilled glass slide. The slide was air dried in dark and stained with 600 nM DAPI for 20 min. A minimum of 50 chromosome spread were counted.

### Statistical analysis

Statistical analysis was performed in either GraphPad Prism or R package software. For direct comparisons of two datasets of values two tailed Mann–Whiney *t* test were performed. For chromosome counts, two tailed Kolomogorov-Smirnov test was performed. All data was reported as mean and standard deviation (SD). The number of replicate determinations is provided in the figure legend for each experiment.

### PP6C-AURKA-PLK1 pathway score

For each of the genes *AURKA*, *PLK1*, *PPP2R2A* and *PPP6C*, RNASeq mRNA levels in samples from indicated types of cancer were obtained from The Cancer Genome Atlas (TCGA). Per sample, for each of the four genes, gene expression z-scores were determined based on the mRNA levels of all samples of the same cancer type. Per sample, a continuous pathway score *s* was determined as follows.

$$s = z_{AURKA} + z_{PLK1} - z_{PPP2R2A} - z_{PPP6C}$$

This variable was compared to previously reported scores for chromosomal instability (70-gene CIN70 score^34^) and HRD (230-gene HRD score^35^). Genes whose transcripts are specifically expressed during G1/S, S, G2, G2/M or M/G1 phases of the cell cycle, as previously identified^37^, were removed from the CIN70 and HRD scores 230-gene expression signatures. The new proliferation-adjusted signature scores, respectively denoted CIN70_prolif_adj and HRD_prolif_adj, were independently compared to our cumulative pathway score. In addition, one thousand genes were selected at random and z-scores were calculated as described above. Using these 1000 z-scores, 250 non-overlapping 4-gene signatures were generated, similar to the one generated for above four genes:

$$s_i = z_a + z_b - z_c - z_d\,{\mathrm{with}}\,\{ i \in {\mathbb{Z}}|1 \ge i \ge 250\}$$

Herein, $s_i$ is a gene expression signature score for a sample and $z_a$ to $z_d$ represent the z-scores of genes a–d within the signature. For each sample, the 250 $s_i$ values were calculated. Spearman coefficients (*R*) and *p* values were determined to assess the correlations between the 250 $s_i$ values and HDR scores. One-sample *t* tests were used to assess whether the Spearman *R* and *p* values of our 4-gene signature were statistically significantly different from the 250 random gene signatures.

## Supplementary information

Supplementary Material
