# Supplementary material for: Dysregulated G2 phase checkpoint recovery pathway reduces DNA repair efficiency and increases chromosomal instability in a wide range of tumours
Source: Oncogenesis. 2021 May 15;10(5):41. doi: 10.1038/s41389-021-00329-8 (PMC8124070; doi:10.1038/s41389-021-00329-8)

Fernando et al Supplementary Material

Supplementary Methods

Flow cytometry

Cell cycle phase was assessed by flow cytometry of DNA content as described previously (57). DNA content was analysed on a Flow Analyser Cytoflex 1 (Becton, Dickinson & Company). Cell percentages in G1, S and G2/M phases were determined by partitioning the DNA histogram.

Immunoprecipitation

Cells were released from a 2 mM thymidine block for 10 h. Cells were lysed in an immunoprecipitation lysis buffer containing 150 mM KCl, 5% Glycerol, 10 mM $MgCl_2$, 0.5 mM EDTA, 0.05% IGPAL protease and phosphatase inhibitors. Lysates were incubated with anti-PLK1 antibody pre-immobilized onto protein G Sepharose beads overnight at 4°C. Immunocomplexes were precipitated, washed and eluted by boiling. Immunoprecipitated proteins were analysed for pPLK1 and PLK1 by immunoblotting.

Detergent soluble and chromatin-associated fractionation

Soluble cytoplasmic and nuclear fractions were prepared using a lysis buffer containing 100 mM NaCl, 20 mM Tris-HCl pH 7.4, 1 mM EDTA, 0.5% NP40, 5mM MgCl2, 250 mM Sucrose with protease and phosphatase inhibitors for 30 min at 4°C. Detergent soluble cytoplasmic supernatant was collected by centrifuging. The residual pellet was washed then extracted with lysis buffer containing 300 mM NaCl, 20 mM Tris-HCl pH 7.4, 1 mM EDTA, 0.5% NP40,

0.5% SDS with protease and phosphatase inhibitors for 30 min. The lysate was centrifuged and the supernatant containing chromatin-associated protein was collected.

Supplementary Figure Legends

Supp. Figure S1. [A] Empty vector (EV) and AURKA overexpressing cells were treated with 2 mM thymidine for 24 h to arrest cells in G1/S phase or treated with 2 μM nocodazole overnight to produce a mitotically arrested population. Cell lysates were immunoblotted with indicated antibodies, phospho-AURKA T288 is pAURKA. α-tubulin was used as a loading control. [B, C] Representative line graphs showing the percentage of cells in G1, S and G2/M phases quantified based on DNA content. A2058 [B] and A375 [C] cells were synchronised with thymidine for 24 h then released into fresh media, harvested at every 2 h interval, analysed for DNA content by FACs. [D] Time in mitosis was assessed from time lapse imaging of EV and AURKA over-expressing controls. The data are from >100 mitotic cells.

Supp. Figure S2. [A] A375 EV and AURKA overexpression cells treated with either 5μM of ICRF193 or DMSO control, were imaged by time-lapse microscopy. The linear regions of the cumulative mitosis curve was used to generate a slope, and this was used as measure of the rate of progression into mitosis. The steeper the slope, the larger the value for the slope and the faster the rate of progression. The arrow represents the slope of the cumulative mitotic index curve. The bar graph shows G2 phase delay assessed based on cumulative mitotic index. . Bars represent means (± SD); $**p < 0.01$. [B] Immunoblot analysis for phospho-CHK2 Thr68 (pCHK2) levels in A2058 and A375 isogenic cell lines treated with either ICRF193 (5μM) or etoposide (2μM) or DMSO control, for 6 h. α-tubulin was used as a loading control

Supp. Figure S3. ATM checkpoint functions are defective in PPP6C mutant melanomas

D35 PP6C mutant melanoma cells were treated with 5µM of ICRF193 and imaged by time-lapse microscopy. Cumulative mitotic index was assessed.

Supp. Figure S4. [A] Immunofluorescence visualization of pPLK Thr210 and DNA (DAPI) in untreated A375 EV and AURKA cells. [B] Dot plot of the quantification of immunofluorescence staining for pPLK1 intensities in asynchronously growing A2058 cells treated with the AURKA/B inhibitor for MLN8237 (alisertib; 1µM) or selective AURKAi (MK5108; 1µM) for 4h, or DMSO. Intensities were quantified using high content imaging. Bars represent means (± SD); ****$p < 0.0001$. [C] A2058 AURKA-overexpressing cells were treated with 2 µM nocodazole overnight or, [D] A2058 EV and AURKA-overexpressing cells were released from a thymidine synchrony block treated with or without ICRF193 and harvested after 10 h. Cell lysates were immunoprecipitated using a PLK1 antibody and immunoblotted for PLK1 and pPLK1 T210. Lysate of nocodazole arrested mitotic cells is shown as a control for pPLK1 (Noco).

Supp. Figure S5. The indicated cell line was treated with or without etoposide for 24 h then the DNA content analysed by FACS.

Supp. Figure S6. [A] A2058 cells were treated with or without etoposide (Etop) for 24 h then with or without ATMi for 4 h. Cells were harvested and immunoblotted for activated CHK1 (pCHK1 S317), CHK2 (PCHK2 T68) and α-tubulin as a loading control. [B] Immunoblot

analysis for pCHK1 S317 levels of A2058 and A375 isogenic cells after UV irradiation (150 J/m2). α-tubulin was used as a loading control.

Supp. Figure S7. Asynchronously growing A2058 and A375 cells were transfected with control siRNA then treated with 2μM etoposide, 500nM BI2536 alone or in combination. G2 phase delay was quantified using time lapse imaging.

Supp. Figure S8. A375 EV and AURKA overexpressing cells were exposed to 6 Gy irradiation and harvested immediately after or 6 h later. [A,B] γ-H2AX intensities were quantified in G1 and S/G2 cell cycle phases as in Figure 6C,D. [C] A2058 and A375 EV and AURKA overexpressing cells were co-stained for Cyclin A and the intensity of Cyclin A and DNA content for each cells was assessed by high content imaging as above. The percentage of cells in G1 and S/G phases are shown for each population.

Supp. Figure S9. [A] S/G2 phase γ-H2AX intensities were quantified in ATM defective SKMEL13 and PP6C mutant D35 melanoma cell lines. [B] S/G2 phase γ-H2AX intensities were quantified in PP6C mutant C025 melanoma cell line. [C] The percentage of S/G2 phase cells based on Cyclin A staining assessed by high content imaging for the two cell lines is shown. Bars represent means of at least three replicates (± SD).

Supp. Figure S10. [A] Immunofluorescence images for γ-H2AX (blue) and Rad51 foci (red) in A375 EV and AURKA overexpression cell lines 6 h after γ–irradiation (10 Gy). [B] The number of RAD51 nuclear foci were counted in the indicated control and irradiated cell lines. 100 cells were counted for each condition. [C] A2058 EV and AURKA overexpression cells were harvested immediately after 6 Gy irradiation or 6 h later. High-salt soluble and non-soluble chromatin associated subcellular fractions were extracted from lysates and immunoblotted against Rad51. [D] γ-H2AX and 53BP1 foci formation in A375 cells 6 h after 10 Gy irradiation. [E] The number of 53BP1 nuclear foci were counted in the indicated control and irradiated cell lines. 100 cells were counted for each condition. [F] γ-H2AX and Rad51 foci formation in10 Gy irradiated A375 cells either pre-treated with the ATM inhibitor 10μM KU55933 or DMSO.

Supp. Figure S11. Scatter plots comparing the *AURKA*, *PLK1*, *PPP2R2A* and *PPP6C* 4-gene signature to gene expression signatures for chromosomal instability (CIN70) and homologous recombination defects (HDR). *R* and *p* values: Spearman correlations. Abbreviations for cancer types: BLCA, bladder cancer; BRCA, breast cancer; LIHC, liver hepatocellular carcinoma; LUAD, lung adenocarcinoma; CESC, cervical squamous cell carcinoma; COAD, colorectal adenocarcinoma; ESCA, oesophageal adenocarcinoma; HNSC, head and neck SCC; Liver, Liver hepatocellular carcinoma; LUSC, lung SCC; OV, ovarian cancer; STAD, stomach adenocarcinoma; SKCM, melanoma; UCS, Uterine carcinoma; SARC, sarcomas.

Supp. Figure S12. Spearman *R* and -$\log_{10}$(*p* values) of correlations between 250 random, non-overlapping 4-gene signatures and the HRD scores are shown for ovarian cancer (OV) and skin cutaneous melanoma (SKCM). Blue lines show the median with 95% confidence intervals.

Red lines show the values for the *AURKA*/*PLK1*/*PPP2R2A*/*PPP6C* signature. Spearman *p* values <2.2×10^{-16} were treated as equal to 2.2×10^{-16}. Main *p* values: one-sample *t* tests.

Fernando et al., Supp Figure S1

A

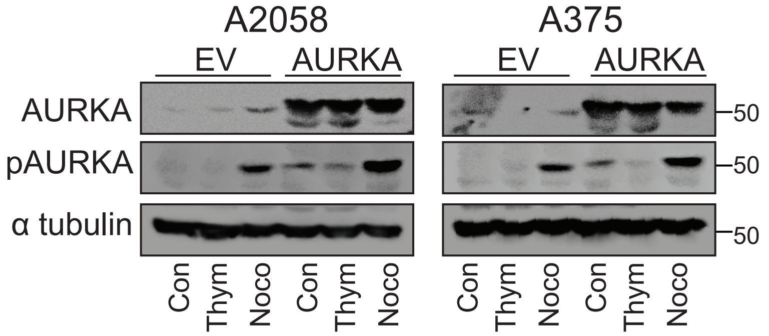

B

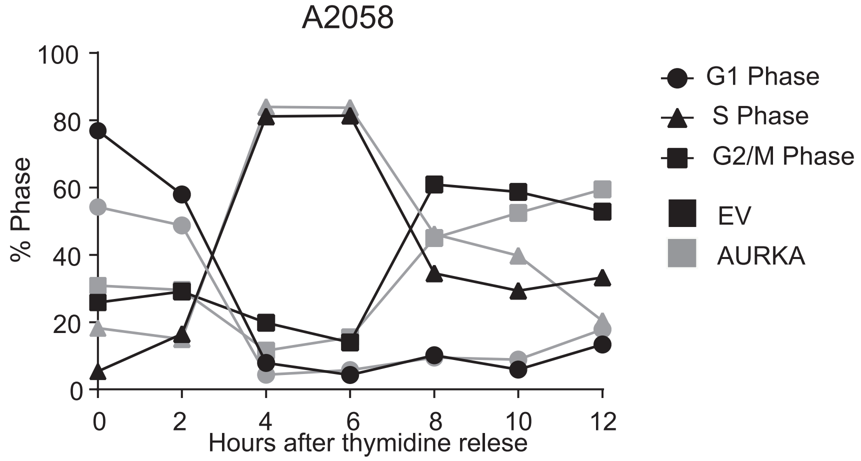

C

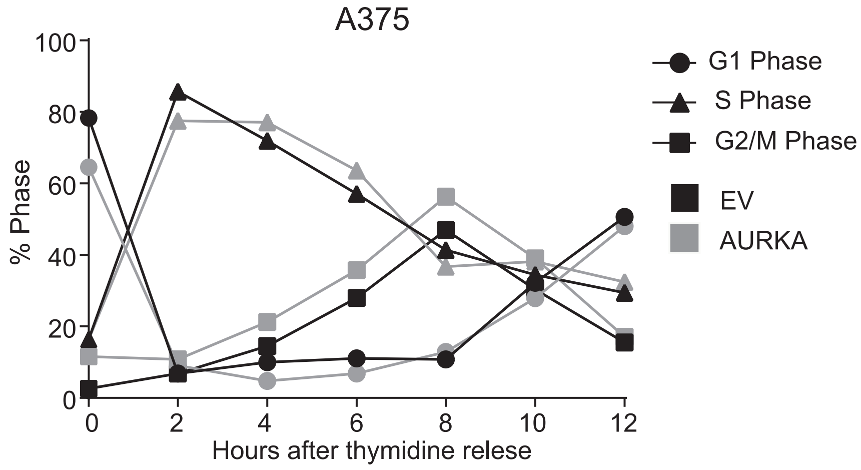

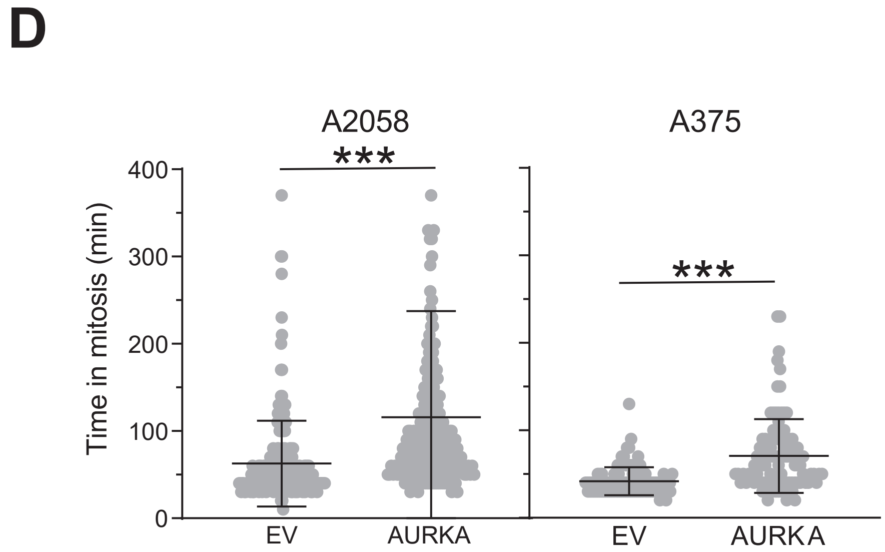
D
A2058
A375
***
***
Time in mitosis (min)
400
300
200
100
0
EV
AURKA
EV
AURKA

Fernando et al., Supp Figure S2

A

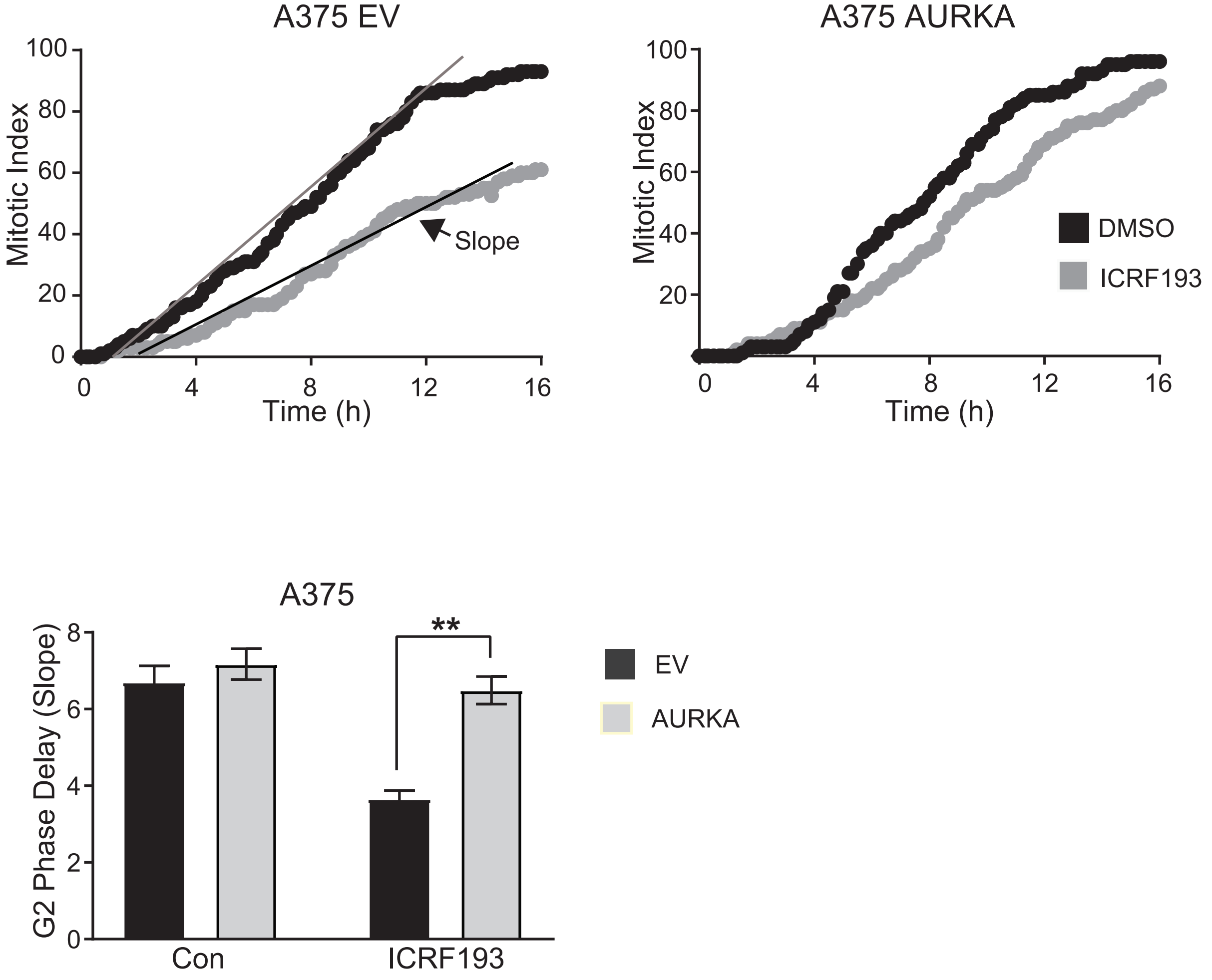

B

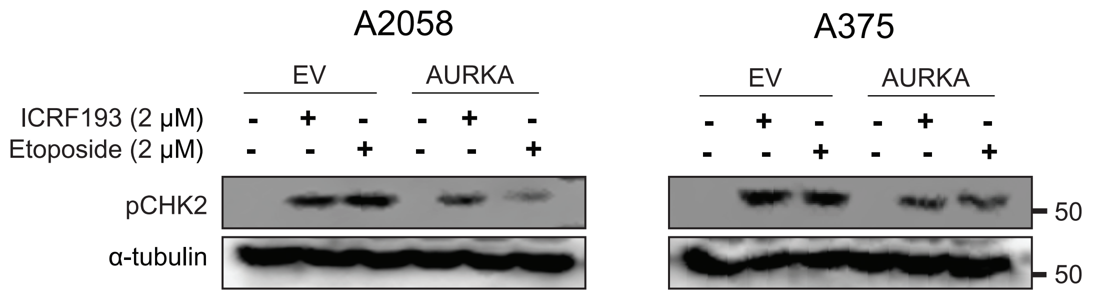

Fernando et al., Supp Figure S3

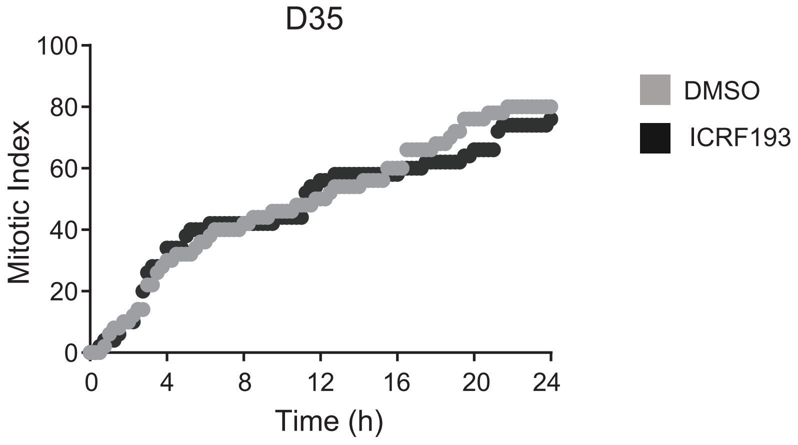

Fernando et al., Supp Figure S4

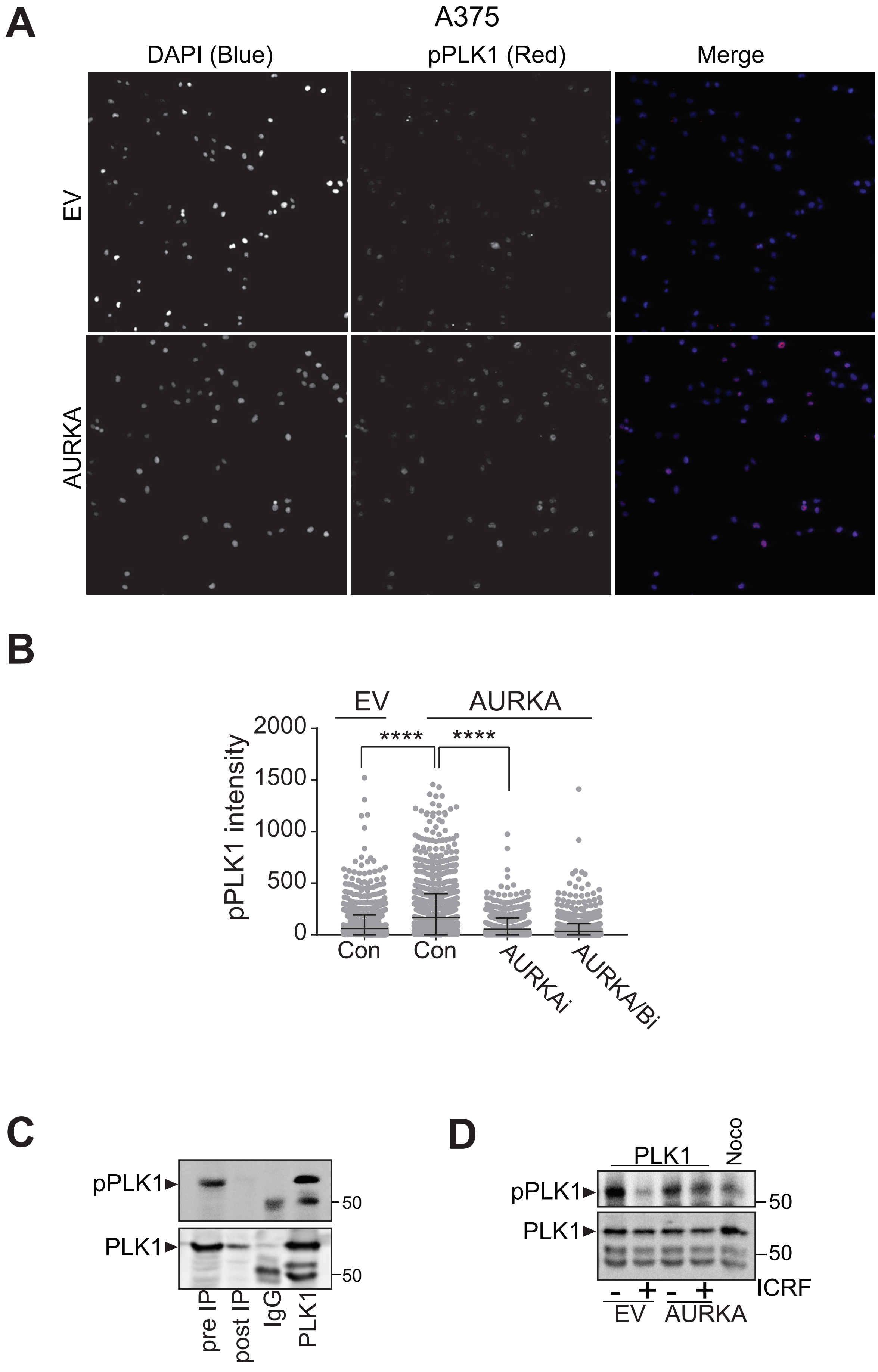

Fernando et al., Supp Figure S5

A2058 EV

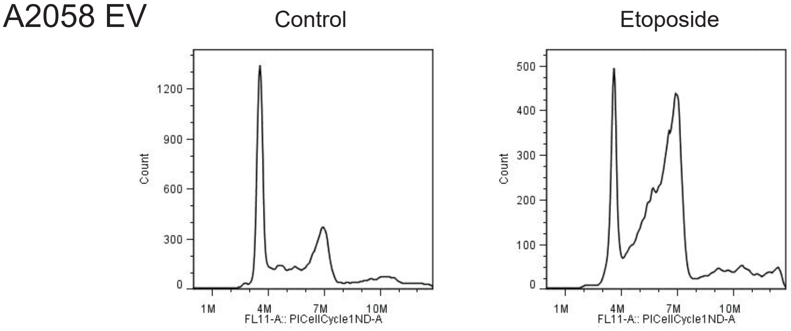

A2058 AURKA

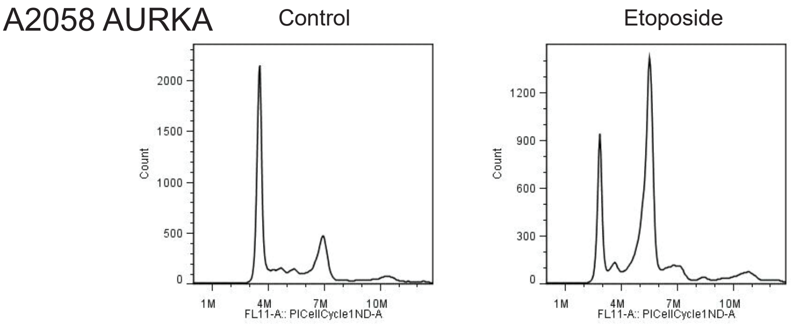

A375 EV

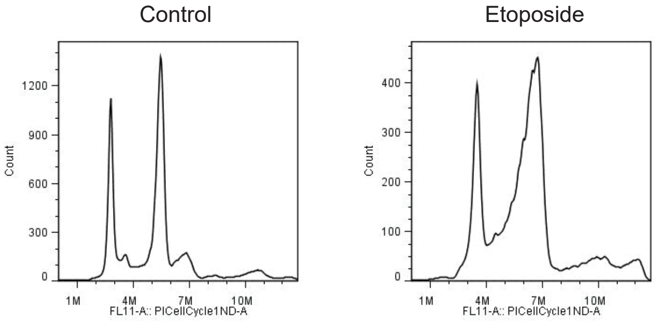

A375 AURKA

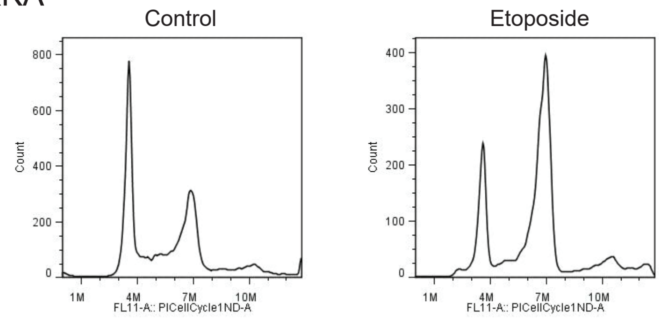

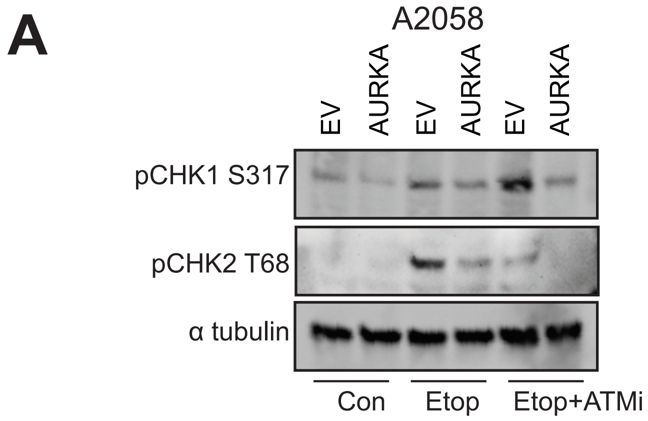
A
A2058
EV
AURKA
EV
AURKA
EV
AURKA
pCHK1 S317
pCHK2 T68
α tubulin
Con
Etop
Etop+ATMi

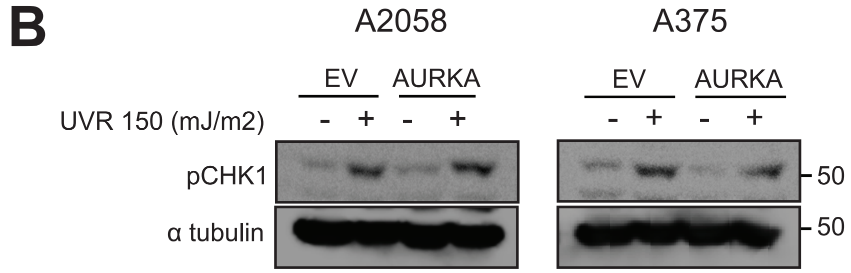
B
A2058
A375
EV
AURKA
EV
AURKA
UVR 150 (mJ/m2)
- + - +
- + - +
pCHK1
α tubulin
50
50

Fernando et al., Supp Figure S7

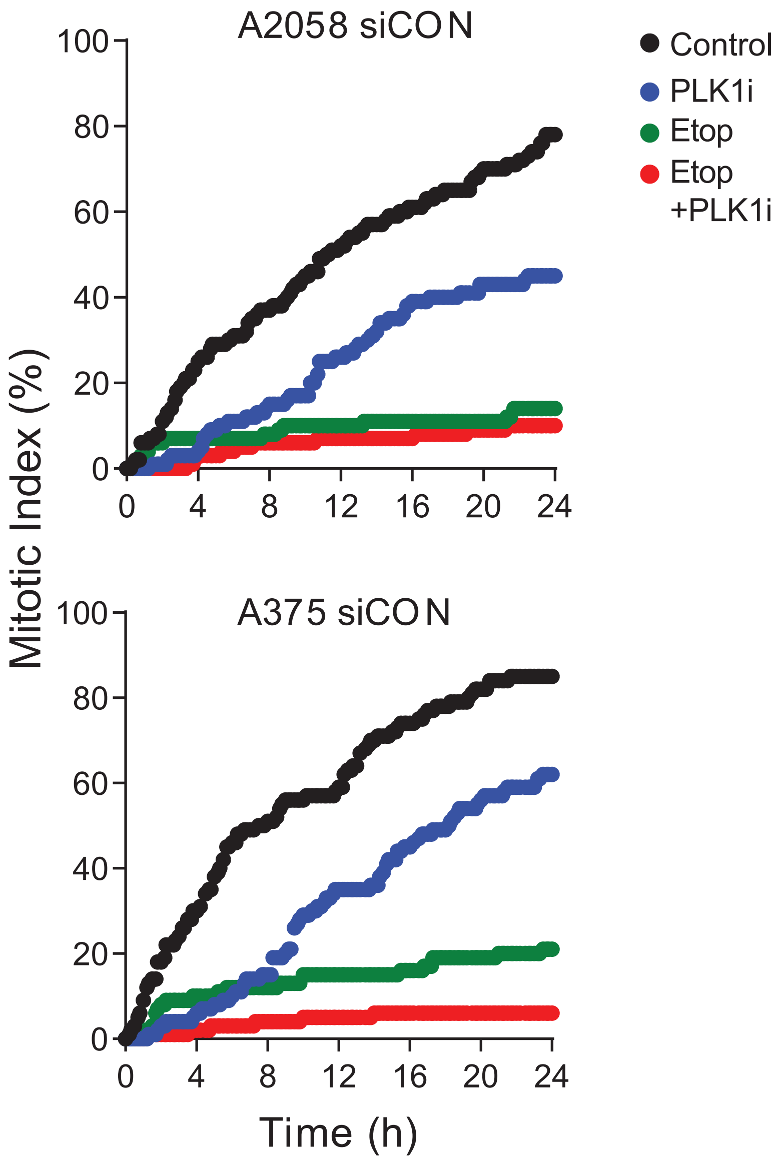

Fernando et al., Supp Figure S8

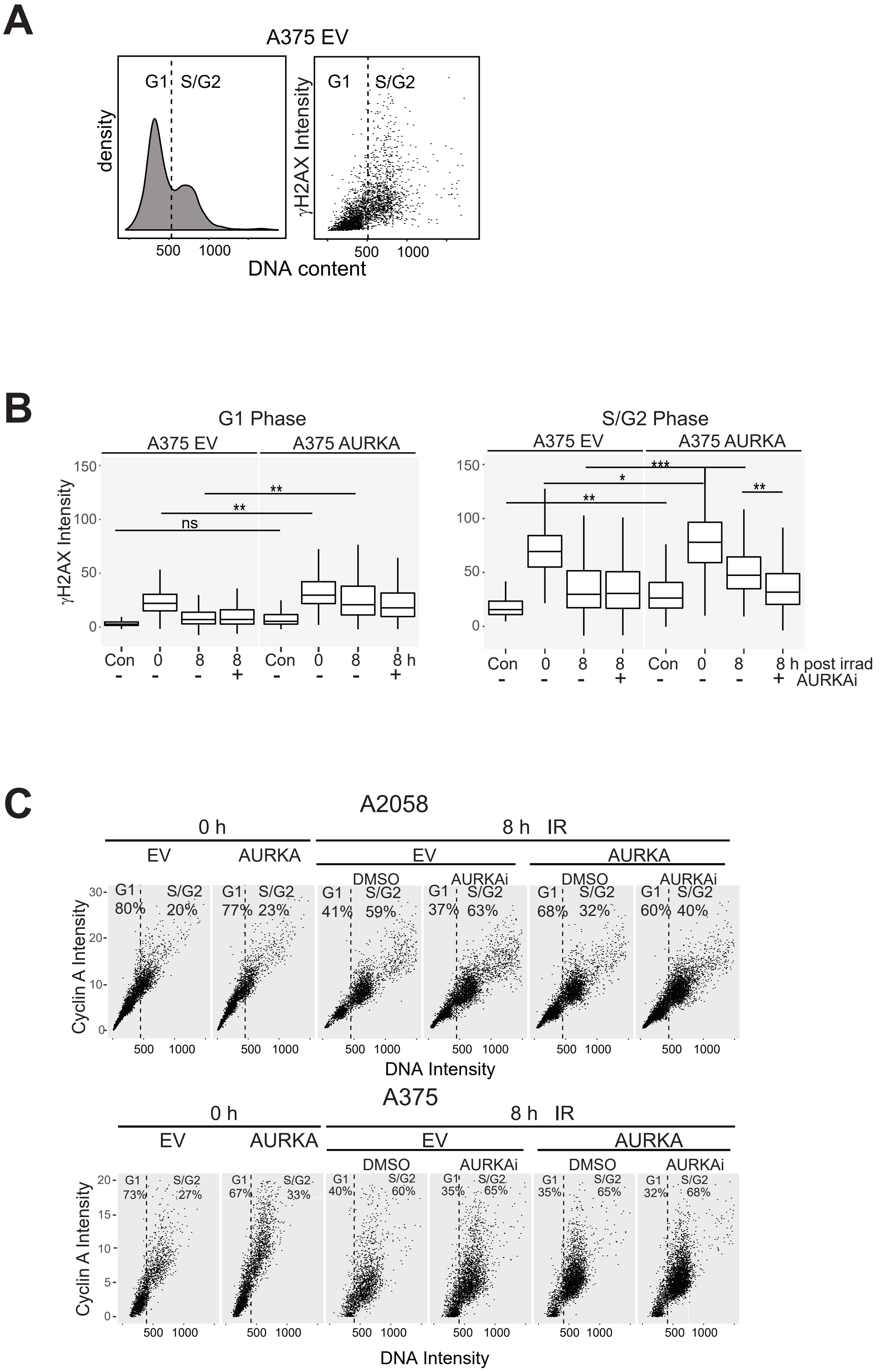

Fernando et al., Supp Figure S9

A

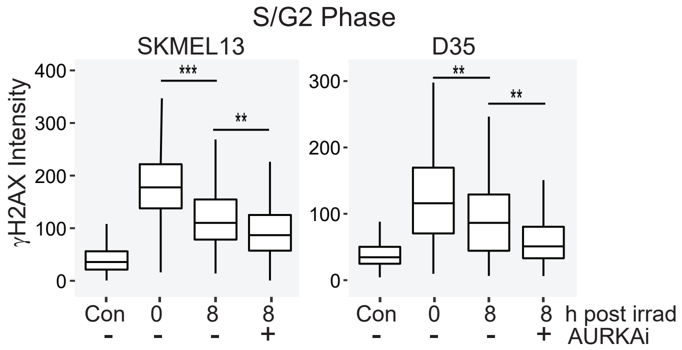

B

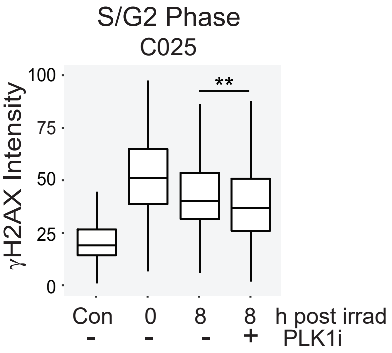

C

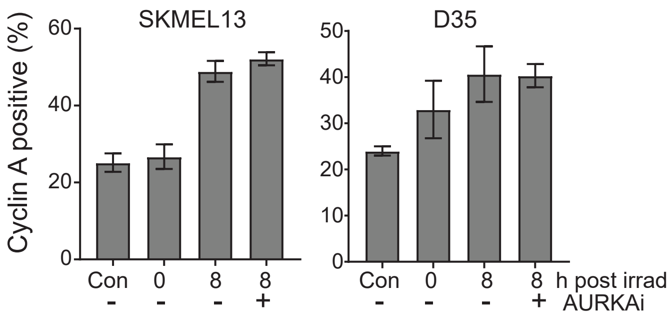

Fernando et al., Supp Figure S10

A

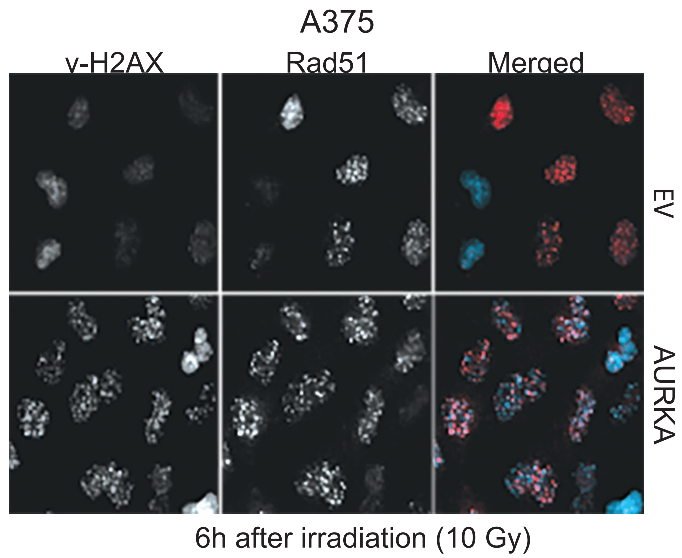

B

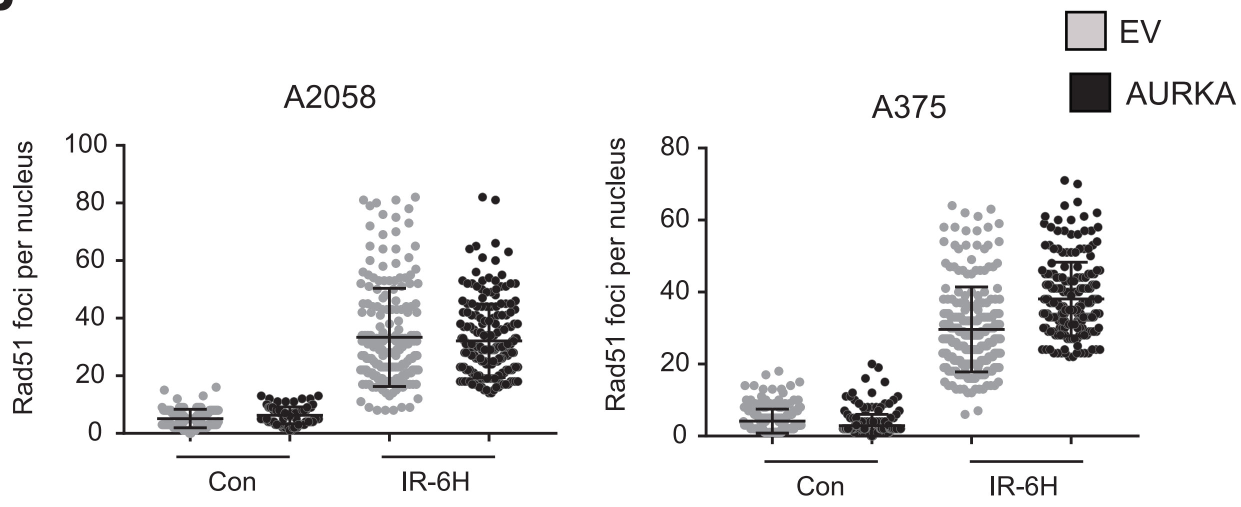

Fernando et al., Supp Figure S10

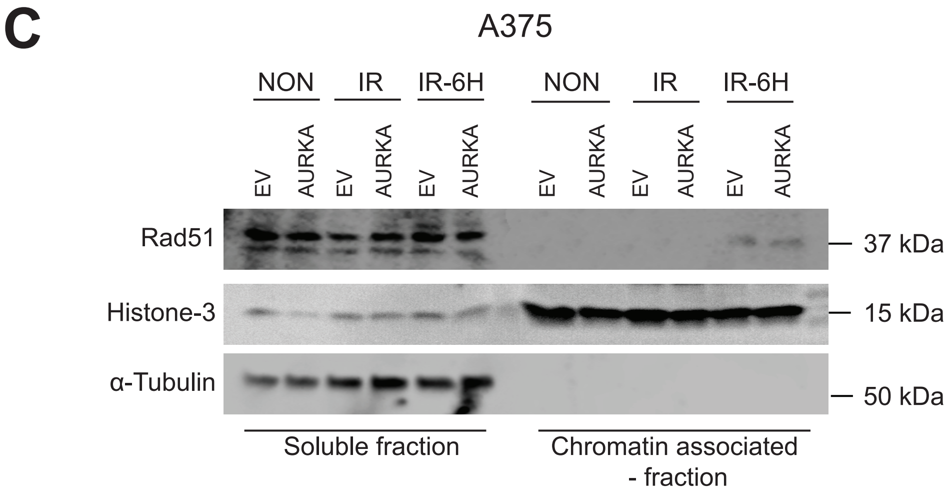

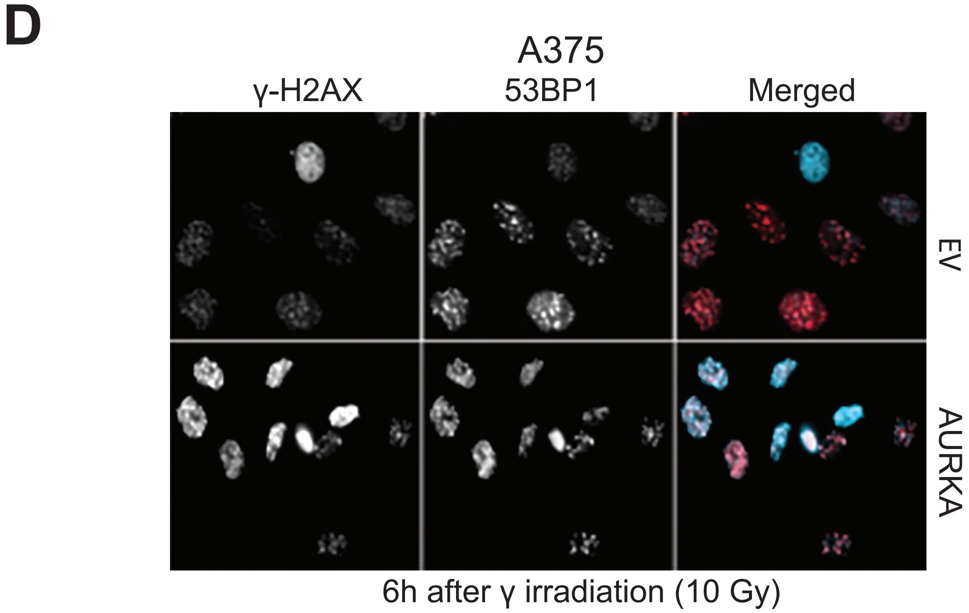

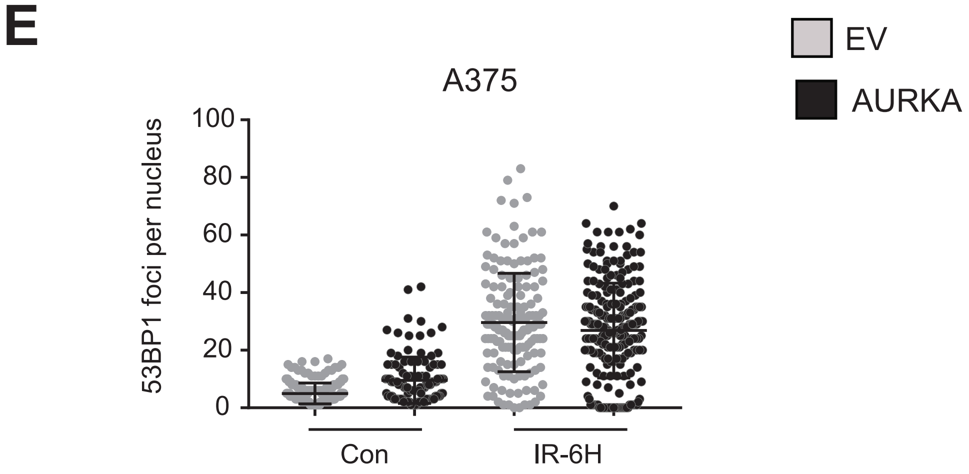

Fernando et al., Supp Figure S10

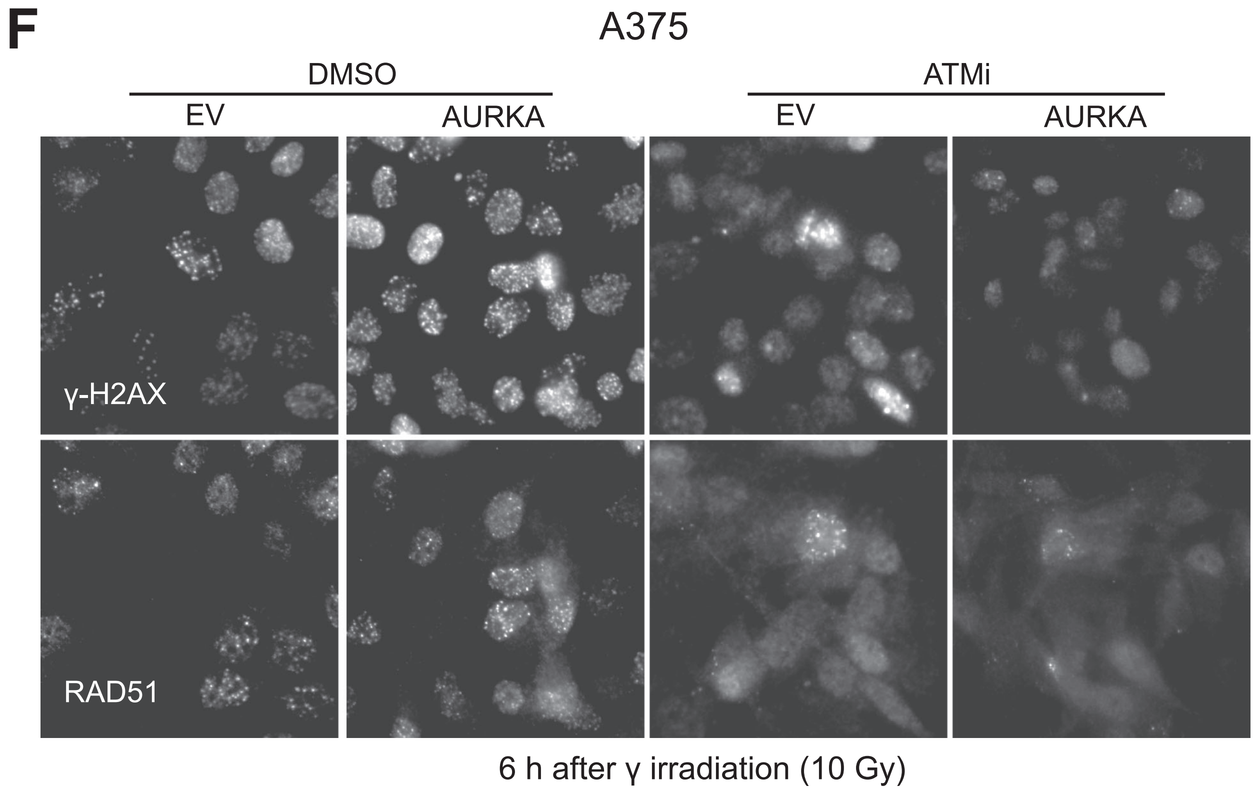

Fernando et al., Supp Figure S11

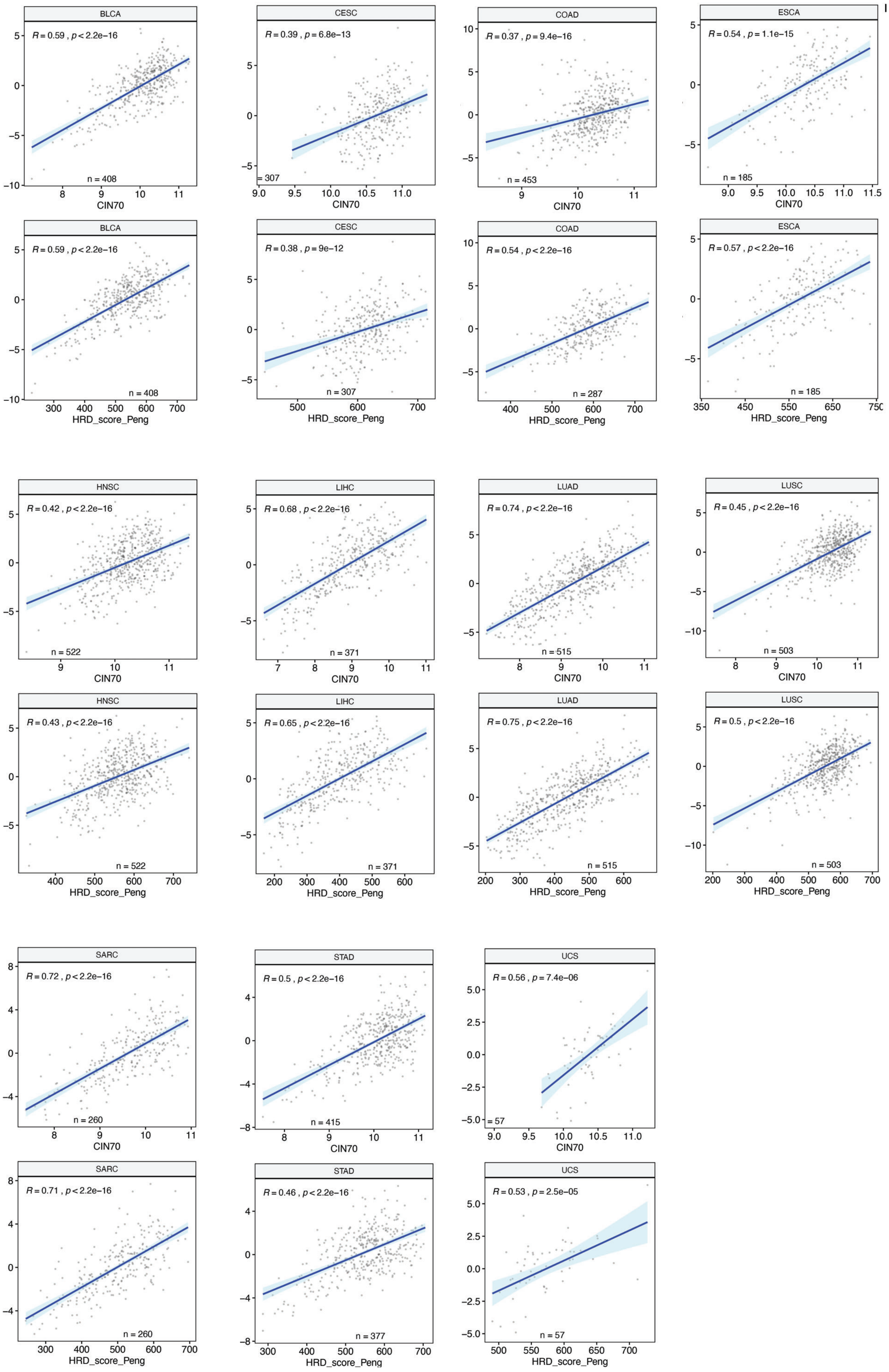

Fernando et al., Supp Figure S12

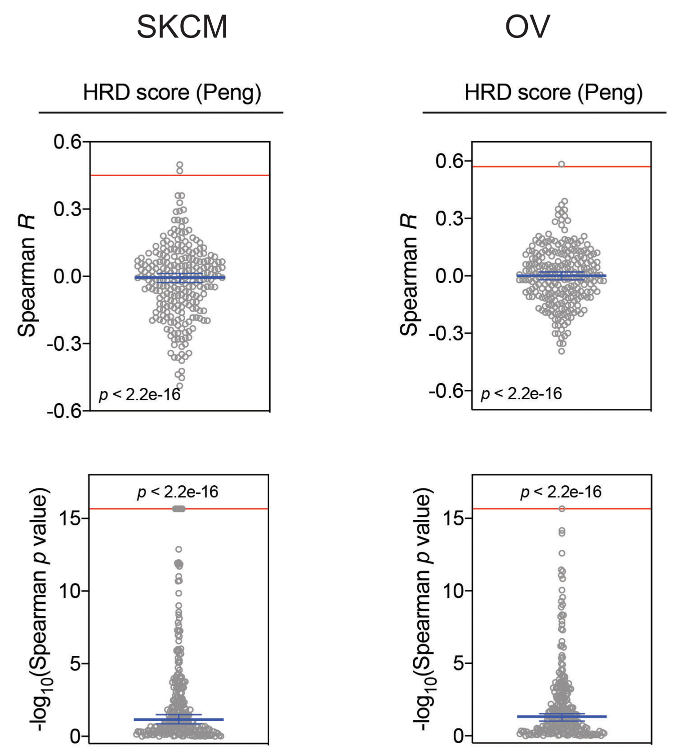

Supplement: Supplementary file 1 — Supplementary Material [file 41389_2021_329_MOESM1_ESM.pdf]
